# ACRC/GCNA is an essential protease that repairs DNA–protein crosslinks during vertebrate development

**DOI:** 10.1093/nar/gkag324

**Published:** 2026-04-20

**Authors:** Cecile Otten, Marin Kutnjak, Christine Supina-Pavic, Marija Pranjic, Ivan Anticevic, Vanna Medved, Marta Popovic

**Affiliations:** Division for Marine and Environmental Research, Ruđer Bošković Institute, Bijenička cesta 54, 10000 Zagreb, Croatia; Division for Marine and Environmental Research, Ruđer Bošković Institute, Bijenička cesta 54, 10000 Zagreb, Croatia; Division for Marine and Environmental Research, Ruđer Bošković Institute, Bijenička cesta 54, 10000 Zagreb, Croatia; Division for Marine and Environmental Research, Ruđer Bošković Institute, Bijenička cesta 54, 10000 Zagreb, Croatia; Division for Marine and Environmental Research, Ruđer Bošković Institute, Bijenička cesta 54, 10000 Zagreb, Croatia; Division for Marine and Environmental Research, Ruđer Bošković Institute, Bijenička cesta 54, 10000 Zagreb, Croatia; Division for Marine and Environmental Research, Ruđer Bošković Institute, Bijenička cesta 54, 10000 Zagreb, Croatia

## Abstract

DNA–protein crosslinks (DPCs) are toxic DNA lesions that block all DNA transactions including replication and transcription, and the consequences of impaired DNA–protein crosslink repair (DPCR) are severe. At the cellular level, impaired DPCR leads to the formation of double strand breaks, genomic instability, and cell death, while at the organismal level, it is associated with cancer, aging, and neurodegeneration. Despite its importance, the mechanisms of DPCR at the organismal level are largely unknown. Proteases play a central role in DPCR, as they remove proteinaceous part of the DPCs, while the peptide remnant crosslinked to DNA is subsequently removed by other repair factors. We characterized the role of putative protease ACRC/GCNA (ACidic Repeat Containing/Germ Cell Nuclear Antigen) in DPCR at the organismal level. For this purpose, we have created new animal models with CRISPR/Cas system: two zebrafish lines with inactive Acrc. We were able to overcome the early embryonic lethality caused by Acrc inactivation by injecting Acrc-WT messenger RNA and have created a viable animal model to study the role of Acrc in adult tissues. We identified histone H3, topoisomerases 1 and 2, Dnmt1, Parp1, Polr3a, and Mcm2 as putative DPC substrates of Acrc. We have shown that Acrc is essential for vertebrate development, and that the mechanism behind it is DPC removal.

## Graphical Abstract

**Graphical Abstract:**
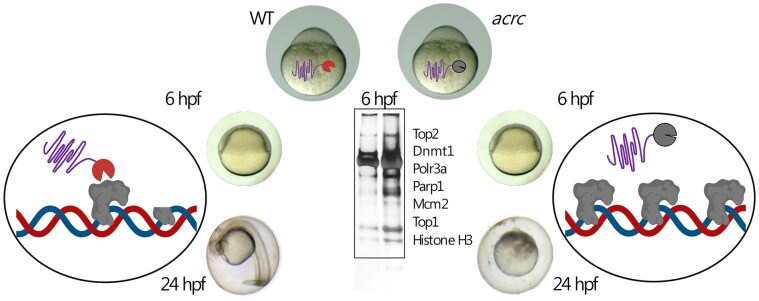

## Introduction

DNA–protein crosslinks (DPCs) are the second most common DNA lesions in cells [1]. They occur endogenously at high frequency (6000 DPCs per cell daily) and are induced by byproducts of normal cellular processes such as reactive oxygen species, formaldehyde, and helical DNA changes [2, 3]. External sources of DPCs include ultraviolet and infrared radiation, as well as various chemicals present in the environment, including chemotherapeutics and transition metals [1]. Due to their bulkiness, DPCs can stall replication and transcription machineries, ultimately leading to accumulation of mutations and/or cell death. Defective repair of DPCs can lead to aberrations during replication, resulting in genome instability, which in turn can lead to cancer or neurodegeneration [4]. DPCs are highly heterogenous and vary by the size of the protein or protein complex, the chemical nature of the crosslink and the DNA topology in the vicinity of the crosslink (intact DNA, double or single strand break). The main DPC repair pathways are (i) proteolysis followed by removal of the peptide remnant from the DNA backbone by the action of nucleotide excision repair or the tyrosyl–DNA phosphodiesterases TDP1 and TDP2 and (ii) the nucleolytic pathway, by which the part of the DNA to which the crosslinked protein is bound is excised [2, 5, 6].

The first DPCR protease, Wss1 (Weak Suppressor of Smt3) was identified in yeast in 2014 [7] followed by SPRTN (SprT-Like N-Terminal Domain), its functional homolog in metazoans [8–11]. Both proteases belong to zinzin family of metallopeptidases and share a highly conserved protease core (HEXXH) with a catalytic glutamate and three zinc-binding histidines, within the SprT-like domain. SPRTN is an essential protease as its knock-out in mouse causes early embryonic lethality [11]; it is specifically active during replication and is very pleiotropic, i.e. can proteolyse various DPCs including histones, topoisomerases 1 and 2 (TOP1 and TOP2), high mobility group protein 1 (HMG1), helicase like transcription factor (HLTF), and Fanconi-associated nuclease 1 (Fan1) [8–10, 12, 13]. Other proteases which have been recently indirectly implicated in DPCR, but lacking a SprT-like domain, are FAM111A, which interacts with Proliferating Cell Nuclear Antigen (PCNA) and plays a role in the resolution of Topo-1cc and trapped PARP-1 [14, 15], and DNA Damage Inducible 1 (DDI1/Ddi1) and 2 (DDI2/Ddi2) which interact with the proteasome and promote replication fork restart after replication stress [16–21]. These proteases, like SPRTN, are also specifically active during replication.

Interestingly, the acidic repeat-containing protein also known as germ cell nuclear antigen (ACRC/GCNA) also harbors a SprT-like domain carrying HEXXH active site [4, 22] and has been linked to the repair of topoisomerase 2 (TOP2)-DPCs and DNA-methyltransferase 1 (DNMT1)-DPCs [23–25]. However, direct evidence of the ACRC protease activity is still lacking, as well as the molecular characterization of its role in DPCR. Given the paucity of knowledge about DPCR on the organismal level and the role of ACRC in DPCR, we turned to zebrafish (*Danio rerio*), a well-characterized vertebrate model organism, to determine the function of Acrc *in vivo*, as well as to biochemically characterize its domains and role in DPCR. Previous studies have shown that ACRC/GCNA plays a role in germ cells: Gcna-1 dysfunction in *Caenorhabditis elegans* resulted in germline lethality [23] while *gcna* mutants in *Drosophila* and zebrafish showed maternal-effect lethality due to chromosome segregation defects in oocytes [24, 25].

ACRC is an evolutionarily ancient protein that typically harbors four domains: an intrinsically disordered region (IDR), a zinc finger, and an HMG box for DNA binding, and the SprT-like domain, a zinc-metalloprotease domain with a characteristic HEXXH catalytic core [2, 22]. Interestingly, the mouse ortholog of ACRC lacks the SprT-like domain and consists almost entirely of the IDR. Male *Acrc* knock-out mice are sterile, suggesting an important role for the IDR domain in mouse male fertility [22, 25, 26]. In humans, defective DPCR proteases have been associated with cancer: mutations in *SPRTN* cause a Ruijs–Aalfs syndrome characterized by progeria and early onset liver cancer due to genomic instability resulting from DPC accumulation [8, 27, 28]. Mutations in *ACRC* have been associated with pediatric germ cell tumors [25] and spermatogenic failure and male infertility [29].

In order to investigate the role of ACRC in DPC repair we have created a separation-of-function mutant zebrafish strain with a mutation in the protease core of Acrc. We show that this catalytic mutation causes early onset embryonic lethality, thus suggesting that the proteolytic function of ACRC is essential in early vertebrate development. We confirmed that this effect is specific with a series of rescue experiments where we inject Acrc-WT messenger RNA (mRNA) into the mutant zygotes which resulted in a complete rescue, i.e. viable embryos, while the injection of the Acrc-E451A mRNA (catalytic mutation) could not overturn lethality phenotype. Mouse Acrc, which lacks a SprT-like domain, could also not rescue the lethality phenotype and neither could Sprtn protease. We then characterized different domains of Acrc, including Sprt domain, IDR region, and C-terminal part and found that intact Sprt domain is essential for the function of Acrc in the early vertebrate development. Using biochemical analyses of the crosslinked proteins we further show DPCs hugely accumulate before the onset of lethality, suggesting that DPC overload due to the Acrc deficiency is the cause of early embryonic lethality. We detected a significant increase in high molecular weight (HMW) DPCs (HMW-DPCs), followed by crosslinked proteins of medium molecular weight (MMW) and low molecular weight (LMW). Specifically, high accumulation of toposiomerases 1 and 2 (Top1-, Top2-) and Parp1-DPCs and significant accumulation of histone H3-, Dnmt1-, Polr3a-, and Mcm2-DPCs in Acrc mutant fish strongly suggest that latter proteins are newly identified DPC targets of Acrc. Furthermore, by injecting Acrc-WT mRNA in homozygous mutant strain we have been able to compensate for the need of Acrc in the early vertebrate development, and the adult fish are viable. In this way, we have created a viable animal model to study the role of Acrc in adult organism. We show that human and zebrafish Acrc share one-to-one orthology, conserved synteny and domain organization. We also show that *acrc* is highly expressed across adult tissues, with predominant expression in gonads. The consequences of the impaired Acrc activity in the adult tissues remain to be determined. Our results demonstrate the importance of Acrc for DPC repair at the organismal level and the importance of the protease core for Acrc function in early vertebrate development. Furthermore, we demonstrate that besides SPRTN protease, there is another essential metalloprotease which can repair a wide range of DPC substrates and has potential therapeutic implications, especially given the fact that it is involved in the repair of topoisomerase- and Parp1-mediated DNA damage.

## Materials and methods

### Phylogeny and syntenic analysis

Nucleotide and protein sequences were retrieved from the NCBI (http://www.ncbi.nlm.nih.gov/) database using Blastx algorithm. Sequences were aligned with MUSCLE algorithm [30] and phylogenetic tree was constructed using Maximum Likelihood method in PhyML 3.0.1 software [31]. Conserved synteny analysis between zebrafish and human ACRC was made using Genomicus (http://www.genomicus.biologie.ens.fr/genomicus), a conserved synteny browser synchronized with genomes from the Ensembl database [32]. Multiple sequence alignments used for phylogenetic analysis are provided as Supplementary Data (Supplementary File 1) and are available at Figshare under DOI: 10.6084/m9.figshare.30884879.

### Structural modeling, and domain and protein stability predictions

Models were build using Colabfold (Alphafold) [33–35] and analyzed and visualized with UCSF Chimera [36]. Domains and motifs were identified using SAPS and Motif Scan workspace [37–39]. Protein topology schematics was visualized using IBS software [40]. Disorder probability was predicted using PONDR-FIT [41]. FoldX was used to predict the thermodynamic stability effects of protein mutations and structural changes by decomposing free energy into specific molecular interaction components [42].

### Polymerase chain reaction (PCR) and quantitative PCR (qPCR)

Quantification of zebrafish gene expression was performed using the qPCR method of relative quantification in technical triplicates on 10 ng complementary DNA (cDNA) per reaction using the following primers: to detect zebrafish *sprtn* expression: DrSprtn_qPCR_F (5′-ATTCCCTTCAGTGGCAGAGG-3′) and DrSprtn_qPCR_R (5′-GAGGTTCTGGTGGCGCTTTA-3′) – 90% primer efficiency; to detect zebrafish *acrc* expression: DrAcrc_qPCR_F (5′-ACCCAAACCACAACGTCCTT-3′) and DrAcrc_qPCR_R (5′-ACTGGCGTGTGGATTACAGG-3′) – 90% primer efficiency; to detect the expression of the zebrafish reference gene *eef1a1l1*: DrEF1a_F (5′-TGATGCCCTTGATGCCATTCT-3′) and DrEF1a_R (5′-CACGACCCACAGGTACAGTT-3′) – 91% primer efficiency.

Quantification of mouse gene expression was performed using the qPCR method of relative quantification in technical triplicates on 10 ng cDNA per reaction using the following primers: to detect mouse *ACRC* expression: MmAcrc_qPCR_F (5′-AAAGGCTAGCTGTGTCGTGA-3′) and MmAcrc_qPCR_R (5′-TGTCAGAAGTCTCATCTCCACTG-3′) – 99.8% primer efficiency; to detect the expression of the mouse reference gene *RPLP0*: MmRplp_F (5′-CTGCACTCTCGCTTTCTGGA-3′) and MmRplp_R (5′-TGATGATGGAGTGTGGCACC-3′) – 94.6% primer efficiency.

Expression of target genes was normalized to the reference gene (R) *elongation factor 1α (eef1a1l1*) as previously described [43]. Mouse *Acrc* expression was normalized to the reference gene Ribosomal Protein Lateral Stalk Subunit P0 (*Rplp0)*. qPCR was performed on a 7300 Real-Time polymerase chain reaction (PCR) System (Applied Biosystems) using Power SYBR Green PCR Master Mix (#4367659, Applied Biosystems) according to manufacturer’s instructions. After initial denaturation at 95°C for 10 min, 40 amplification cycles were performed with denaturation at 95°C/15 s, annealing and elongation at 60°C/1 min, all together followed by melting curve analysis. Data analysis was performed using the 7500 Fast System sodium dodecyl sulphate (SDS) software (Applied Biosystems) followed by the relative quantification using Q-Gene method [44, 45] which is based on the following formula yielding the mean normalized expression (MNE):


\begin{eqnarray*}
MNE = E{{\left( R \right)}^{Ct\left( R \right)}} / E{{\left( {\textit{gene}} \right)}^{Ct\left( {\textit{gene}} \right)}},
\end{eqnarray*}

where E (R): amplification efficiency of the reference gene; Ct (R): average Ct value of the reference gene for a particular tissue; E (gene): amplification efficiency of the gene of interest, Ct (gene): average Ct value of the gene of interest for that particular tissue. Data are given as MNE × 10^6^ ± standard deviation (SD).

To verify that the rescue mRNAs are present in the injected embryos, pools of 5–20 embryos were collected at 24 or 48 hours post-fertilization (hpf), total RNA was extracted using the Total RNA isolation kit (#T2010, NEB) following manufacturer’s instructions. Total RNA was then quantified using the Bio-Spec Nano Spectrophotometer and RNA integrity was determined by agarose gel electrophoresis. For each sample, purified total RNA was reverse transcribed using the ProtoScript II First Strand cDNA Synthesis Kit (#E6560, NEB) and a 1:1 mix of oligodT and random hexamer primers to obtain cDNA. Quantification of zebrafish gene expression was performed using the qPCR method of relative quantification in technical triplicates on 5 ng cDNA per reaction using the following primers, with DrAtp5po or 18S used as reference genes: to detect zebrafish *sprtn*: DrSprtnRescue_qPCR_F (5′-AATGACAAGTTCTTCTGGGGG-3′) and DrSprtnRescue_qPCR_R (5′-AAACACCAGCACATAGCGTCA-3′) – 96% primer efficiency; to detect zebrafish *acrc*: DrAcrcRescue_qPCR_F (5′-AGAACCGGTCTCAAGAGGAA-3′) and DrAcrcRescue_qPCR_R (5′- CTCATAGACTGCGGTTGGAC -3′) – 86% primer efficiency; to detect zebrafish *atp5po*: DrAtp5po_qPCR_F (5′-CTTGCAGAGCTGAAAGTGGC-3′) and DrAtp5po_qPCR_R (5′-ACCACCAAGGATTGAGGCAT-3′) – 98% primer efficiency; to detect zebrafish *18S*: Dr18S_qPCR_F (5′-CGCGAGATGGAGCAATAACA-3′) and Dr18S_qPCR_R (5′-AGGGTAGGCACACGTTGAT-3′) – 83% primer efficiency.

Similarly, to verify the expression of the MmACRC rescue construct injected into zebrafish embryos, qPCR was performed using the same primers as described above (MmAcrc_qPCR_F 5′-AAAGGCTAGCTGTGTCGTGA-3′ and MmAcrc_qPCR_R 5′-TGTCAGAAGTCTCATCTCCACTG-3′ – 99.8% primer efficiency) and run on an agarose gel next to a control RT-PCR using DrRplp0 as a reference gene: DrRplp_qPCR_F (5′-GCCCTGCACAAGAGATTCCT-3′) and DrRplp_qPCR_R (5′-GCAAGAGTTGGGTAGCCGAT-3′) – 82% primer efficiency.

### Zebrafish lines and handling

Adult wild-type (WT) zebrafish of the AB strain are kept at 28.5°C on a 14-h light and 10-h dark cycle under standard conditions [46]. Originally, adult zebrafish of both sexes of the AB strain were obtained from the European Zebrafish Resource Center at the Karlsruhe Institute of Technology (Germany). Zebrafish embryos were collected and kept in E3 medium (5 mM NaCl; 0.17 mM KCl; 0.33 mM MgSO_4_; 0.33 mM CaCl_2_) in Petri dishes at 28.5°C. All handling and experiments were performed in accordance with the directions given in the EU Guide for the Care and Use of Laboratory Animals, Council Directive (86/609/EEC), and the Croatian Federal Act on the Protection of Animals (NN 135/06 and 37/13) under the project license HR-POK-023.

### Microinjections

Microinjections were performed using a microinjection system (Laboratory microinjector – FemtoJet^®^ 4× series – Eppendorf) and needles (Eppendorf™ Femtotips™ Microinjection Capillary Tips). Microinjections in one- to four-cell stage embryos were performed by injecting 1 nl of premixed solutions into the yolk for the following purposes: (i) creating zebrafish *acrc* mutant lines using the CRISPR/Cas9 system, (ii) rescue of the *acrc* mutant phenotype by mRNA injection. Embryos were then maintained in E3 medium under standard conditions at 28.5°C and staged as previously described [47].

### Creation of mutant zebrafish strains using CRISPR/Cas9 system, genotyping

Genetically modified zebrafish lines were created using Cas9 protein (EnGen^®^ Spy Cas9 NLS, #M0646T, NEB) and previously established protocols combining PCR and reverse transcription to generate guide RNAs [48]. To create specific zebrafish *acrc* mutant strains, the following two guides targeting *acrc* exon 12 were designed using the CRISPRscan software [49]: DrACRC sgRNA_1 (5′-GGAGCATAAAGCCTCCAGAA-3′), which resulted in the creation of the rbi8 and rbi9 alleles, and DrACRC sgRNA_2 (5′-GGCCGCATGACACATTTCA-3′), which resulted in the creation of the rbi5 allele.

To introduce *acrc* mutations in exon 12, one-cell stage zebrafish embryos were microinjected with 1 nl of the following solution: guide RNA (180 ng/µl), Cas9 protein (600 ng/µl), and KCl (300 mM). F0 injected embryos were raised and founders were later identified by sequencing of F1 progeny. For genotyping experiments, injected embryos were lysed with 0.5 mg/ml proteinase K (#J63710, AlphaAesar) in digestion buffer (10 mM Tris–HCl, pH 8.5, 50 mM KCl, 0.3% Tween-20) for 3 h at 55°C. PCR and sequencing was then performed using the following primer pair: Geno_acrc_F (5′-ACAGATCGGTTACGGGATAC-3′) and Geno_acrc_R (5′-TTGATGTCATAACTGTGGCA-3′).

Three alleles were selected for further maintenance. Experiments were conducted either with the *rbi5* allele which lacks 4 amino acids in the catalytic site of the SprT-like domain of Acrc (ΔEMCH 451–454) or with the other alleles (*rbi8* and *rbi9*, with 1 nt indel and 11 nt indel creating a premature stop at aa 477 and aa 472, respectively. Both mutant lines, *acrc ^rbi5/ rbi5^* and *acrc ^rbi8/rbi9^*, displayed the same phenotype, similar rescue patterns, and the same DPC accumulation, and were used throughout the paper.

### Rescue experiments: cloning and mRNA *in vitro* transcription

To perform rescue experiments, the WT coding sequence of zebrafish *gcna/acrc* (ENSDART00000169970.2) was amplified by PCR from WT adult testis (AB strain) and cloned into the multicloning site of the pCS2 + HisMyc vector [50] between the XhoI and XbaI restriction sites and verified by sequencing. The vector contains an SP6 promoter, a multicloning site, a polyA tail and a unique restriction site after the polyA tag (KpnI or NotI). We have sub-cloned the full-length human *ACRC* coding sequence (NM_052957.5) from a plasmid we obtained from the Niels Mailand’s group (HsACRC-pAcGFP1-C1) [23] into pCS2 + HisMyc vector. mRNA was synthesized using purified, linearized plasmid by performing an *in vitro* transcription with SP6 using the Hiscribe SP6 RNA kit (#E2070S, NEB) and a cap analog from the ARCA kit (#S1411, NEB) for improved mRNA stability. The resulting mRNA was purified using the RNA cleanup kit (#T2040, NEB) and injected into 1–2 cell stage *acrc* maternal mutant embryos or into control WT embryos (1 nl of the mRNA solution: 250 ng/μl RNA in 0.3M KCl and 0.015% phenol red, except for DrSprtn: 100 ng/μl RNA). To determine functionally relevant amino acids and domains of *acrc*, we cloned deletion constructs by inverse PCR based on the original pCS2 + HisMyc-DrAcrc plasmid using the following primer pairs: pCS2 + HisMyc-Acrc-WT was created using DrAcrc_F (5′-AGAGGATCTGCTCGAGATGGATCCTGGTACTTTATCACT-3′) and DrAcrc_R (5′-TCACTATAGTTCTAGATCAACTTTGACTGAGACGAGTCT-3′); pCS2 + HisMyc- Acrc-E541A was created using DrAcrc-E451Amut_F (5′-GCTATGTGTCATGCGGCC-3′) and DrAcrc-E451Amut_R (5′-ATGGATTAAAGTATCCCGTAACC-3′); pCS2 + HisMyc- Acrc-ΔC was created using DrAcrc-Cterm-del_F (5′-TGATCTAGAACTATAGTGAGTCGTA-3′) and DrAcrc-Cterm-del_R (5′-CAACAAAACCAGCTGTCC-3′); pCS2 + HisMyc- Acrc-ΔSprT was created using DrAcrc-SprT-del_F (5′-TGATCTAGAACTATAGTGAGTCGTA-3′) and DrAcrc-SprT-del_R (5′-TTTGTTCTGTTTGAAACTCCG-3′); pCS2 + HisMyc- Acrc-SprT was created using DrAcrc-SprT_F (5′-GTTGTGTGTAAAACTCCCGG-3′) and DrAcrc-SprT_R (5′-CATCTCGAGCAGATCCTCTTC-3′).

RNAs were then *in vitro* synthesized and injected as described above for WT *acrc*. Similarly, we obtained rescue constructs based on the same pCS2 + HisMyc vector backbone with full-length coding sequences inserted between the XhoI and XbaI restriction sites for mouse *Acrc* (NM_001 382 234) or zebrafish *sprtn* (ENSDART00000158057.2) from Genosphere.

### Analysis of rescue experiments

To assess rescue efficiencies, the morphology of 24 hpf noninjected and injected embryos was assessed and embryos were categorized as follows: (i) “dead”; (ii): “necrotic”: the yolk is intact, but the embryo is completely necrotic; (iii) “very abnormal”: the embryo has a very short body axis due to necrosis and incomplete embryonic development; (iv) “abnormal”: the embryo has a head and an elongated tail but can display defects such as cyclopia, which are typically the result of earlier necrosis; (v) “WT-like”: the embryo has a morphology indistinguishable from WT. Each rescue construct was injected at least two times in separate experiments (different breeding pairs) in *acrc ^rbi5/rbi5^* and/or *acrc ^rbi8/rbi9^*, and at least 15 embryos were categorized per experiment. Results are shown as the percentage of embryos in each category. To better compare the rescue efficiency of the various constructs statistically, we grouped the described phenotypes into “dead” (categories 1 and 2) and “alive” (categories 3, 4, and 5), and determined the percentage of survival in each condition. Raw data from all rescue experiments are available on Figshare under DOI: 10.6084/m9.figshare.30920183.

### Whole-mount immunofluorescence

For each condition, we collected a pool of 15 embryos at 6 hpf in a 1.5 ml tubes and fixed them with 4% paraformaldehyde overnight at 4°C. After a brief wash (1× for 10 min) with PBST (0.1% Triton X-100 in PBS) at room temperature (RT), embryos were manually dechorionated with precision forceps under a binocular and returned to their respective tube. To permeabilize them, embryos were briefly rinsed with water (1×), and incubated at −20°C with 100% ice-cold acetone for 15 min followed by a brief water rinse and a wash (1× for 30 min) with PBST-BD [1% bovine serum albumin (BSA), 1% dimethyl sulfoxide (DMSO) in PBST]. The samples were blocked with 5% normal goat serum in PBST-BD for at least 1 h at RT with gentle rocking. Incubation with anti-myc antibody (9E10, ab-32, Abcam) was performed overnight at 4°C with gentle rocking (1:500 dilution in PBST-BD). Next day, after extensive washes (6× for 30 min) with PBST at RT with gentle rocking, embryos were incubated with a secondary antibody goat anti-mouse IgG H&L (Alexa Fluor 488) (ab-150113, Abcam, 1:500 dilution) and 50 µM 4′,6-diamidino-2-phenylindole (DAPI) in PBST-BD overnight at 4°C with gentle rocking. After extensive washes (6× for 30 min) with PBST at RT with gentle rocking, the yolk of embryos was pierced and gently removed as much as possible, and embryos were flattened and mounted in Vectashield on microscopy slides.

### Imaging and confocal microscopy

Zebrafish embryos were imaged using a Samsung 13-megapixel camera with an f/1.9 aperture applied to the ocular of a Motic SMZ-171 binocular.

For confocal microscopy, slides were examined using a Leica TCS SP8 X confocal laser scanning microscope, equipped with an HC PL APO CS2 40/1.30 oil objective used for higher magnification images, and an HC PL APO CS2 10/0.40 dry objective for lower magnification images. The excitation wavelengths and detection ranges used for imaging were as follows: (i) for DAPI: 405 nm excitation and 410–480 nm detection; (ii) for anti-myc: Alexa Fluor 488 nm excitation and 500–580 nm detection.

### Tissue collection

Adult male and female zebrafish were sacrificed for tissue collection according to regulations by immersion in ice-cold water supplemented with 10% MS-222 (Tricaine) for 30 min and dissected to collect the following tissues: brain, liver, kidney, intestine, gonads (testes and ovaries), eye, gills, muscle. Tissues from *n* = 3 adults were pooled, put in RNALater and frozen. WT zebrafish embryo samples were collected and dry-frozen at 1, 4, 6, 12, 24, 48, and 72 hpf: three pools of 30 embryos per condition. Pools of 10 zebrafish embryos of the following genotype were collected at 6 hpf for total RNA extraction: WT, *acrc^rbi5/rbi5^, acrc^rbi8/rbi9^*. The following mouse tissues were donated by Prof. Tihomir Balog (RBI, see the ‘Acknowledgments’ section): brain, liver, kidney, intestine, gonads (testes and ovaries), and processed similarly to the zebrafish tissues. The mouse tissues were derived from three females and three males, all of them 4 months old.

### RNA extraction and cDNA synthesis

Collected adult zebrafish and mouse tissue samples were thawed on ice and submitted to homogenization at 13 500 min^−1^ for 30 s with Ultra turrax t25. Total RNA was then extracted from max. Then, 50 µg tissue for each sample using the Total RNA isolation kit (#T2010, NEB) following manufacturer’s instructions. Total RNA was then quantified using the Bio-Spec Nano Spectrophotometer and RNA integrity was determined by agarose gel electrophoresis. For each sample, 1 µg of purified total RNA was reverse transcribed using the ProtoScript II First Strand cDNA Synthesis Kit (#E6560, NEB) and random hexamer primers to obtain 50 ng/µl cDNAs.

### Western blot analysis of zebrafish tissues

Zebrafish ovaries (one ovary per sample) were lysed in 500 µl Radioimmunoprecipitation assay buffer (RIPA) buffer [150 mM NaCl, 1% Triton X-100, 0.5% sodium deoxycholate, 50 mM Tris–HCl (pH 8), 0.5% SDS] supplemented with protease inhibitors (leupeptin 1 µg/ml, pepstatin 1 µg/ml, chymostatin 1 µg/ml, aprotinin 10 µg/ml, Phenylmethylsulfonyl fluoride (PMSF) 1 mM) and phosphatase inhibitors (sodium fluoride 2 mM, sodium pyrophosphate 2 mM, sodium orthovanadate 5 mM), followed by homogenization (three cycles of 10 s at medium strength using an Ultra-Turrax T25 homogenizer) and sonication (three cycles of 3 s at low power using an MSE probe sonicator). Lysates were centrifuged (10 000 rcf, 7 min, 4°C), and supernatant was collected for subsequent analysis. Total protein concentration was determined using the Pierce Detergent Compatible Bradford Assay Kit (Thermo Fisher Scientific, #23246). Samples were run on homemade 5%–18% sodium dodecyl sulphate–polyacrylamide gel electrophoresis (SDS–PAGE) gradient gels at 120 V for 90 min using a Mini-PROTEAN 3 Cell electrophoresis chamber (Bio-Rad) and transferred to Polyvinylidene difluoride (PVDF) membranes (Roche, 03010040001) by wet transfer using a Mini Trans-Blot system (Bio-Rad). Membranes were blocked with 5% low-fat milk in TBST buffer [20 mM Tris–HCl (pH 7.5), 150 mM NaCl, 0.1% Tween 20] for 2 h at RT and then incubated with primary antibodies in 2.5% BSA in TBST overnight at 4°C, followed by incubation with horseradish peroxidase (HRP)-conjugated secondary antibodies for 1 h and washing three times for 10 min in TBST. Detection was performed according to the manufacturer’s instructions for the ECL blotting substrate (Bio-Rad, #1705061) and visualized using the ChemiDoc™ XRS + System (Bio-Rad, #1708299). For detection of Acrc and histone H3 (loading control) from tissue lysates, 25 µg of total protein per sample was loaded onto an SDS–polyacrylamide gradient gel. The following primary antibodies were used: anti-ACRC (Sigma–Aldrich, SAB1403429, 1:6000) and anti-histone H3 (Cell Signaling Technology, #9715, 1:3000). HRP-conjugated anti-mouse secondary antibody (Sigma–Aldrich, #A9044, 1:100 000) was used for anti-ACRC, whereas HRP-conjugated anti-rabbit secondary antibody (Sigma–Aldrich, #A0545, 1:100 000) was used for anti-histone H3.

### Isolation and detection of DPCs by SDS–KCl method

Isolation of DPCs was performed using the SDS/KCl precipitation assay as previously described [12, 51]. This method was used for DPC isolation from WT and *acrc^rbi8/rbi9^* maternal mutant zebrafish embryos. Pools of 100 untreated embryos were collected at 6 hpf. Samples were lysed for 15 min at RT with gentle pipetting up and down and occasional gentle vortexing, placed in liquid nitrogen and incubated overnight. Lysis buffer consisted of: 2% SDS, 20 mM Tris–HCl (pH 7.5), protease inhibitors (leupeptin 1 µg/ml, pepstatin 1 µg/ml, chymostatin 1 µg/ml, aprotinin 10 µg/ml, PMSF 1 mM) and phosphatase inhibitors (sodium fluoride 2 mM, sodium pyrophosphate 2 mM, sodium orthovanadate 5 mM). The next day, samples were thawed in a thermoblock (55°C/5 min) and then sonicated (three cycles of 30 s at high power using an MSE probe sonicator). Proteins were precipitated by adding 400 μl KCl buffer [200 mM KCl, 20 mM Tris–HCl (pH 7.5)] followed by incubation on ice for 5 min. The precipitated proteins were pelleted by centrifugation at 21 000 rcf for 5 min (4°C), and the supernatant was kept for quantification of soluble DNA. The pellet was washed three times by adding 400 μl KCl buffer, followed by incubation at 55°C for 5 min, 5 min on ice, and centrifugation at 15 000 rcf at 4°C for 5 min. After washing, each pellet was resuspended in 400 μl KCl buffer containing proteinase K (0.2 mg/ml) and incubated at 55°C for 1 h. After incubation, BSA (1.25 mg/ml) was added and samples were incubated on ice for 5 min. After centrifugation at 15 000 rcf at 4°C for 5 min, the supernatant contained the cross-linked DNA. From both the soluble and cross-linked DNA, 50 μl of the sample was taken for treatment with RNase A (0.2 mg/ml) for 30 min at 37°C. Soluble DNA and cross-linked DNA were quantified using the Quant-iT™ 1× double-stranded DNA (dsDNA) HS assay (Thermo Fischer Scientific, MA, USA), and the amount of DPCs was calculated as the ratio of cross-linked DNA to total DNA (soluble + cross-linked).

### Isolation and detection of DPCs by RADAR method

DPC isolation and detection from zebrafish embryos was performed using the modified RADAR (rapid approach to DNA adduct recovery) assay which we previously adapted [52] from the original protocol developed by Kiianitsa and Maizels [53]. In brief, DPCs were isolated from 50–60 embryos (at 6 hpf) per sample using following steps: (i) lysis with pre-warmed lysis buffer [6 M guanidinium isothiocyanate, 10 mM Tris–HCl (pH 6.8), 20 mM EDTA, 4% Triton X-100, 1% N-lauroylsarcosine sodium, and 5% β-mercaptoethanol] for 10 min at 55°C with vigorous mixing; (ii) addition of an equal volume of 96% ethanol to precipitate DNA with crosslinked proteins; (iii) centrifugation at 10 000 Relative Centrifugal Force (rcf) for 10 min at 4°C; (iv) washing the DNA pellet four times with wash buffer [20 mM Tris–HCl (pH 7.4), 1 mM EDTA, 50 mM NaCl, 50% EtOH] with the same centrifugation steps as in (iii); (v) dissolving the dried pellet in 8 mM NaOH (with sonication: 10 cycles of 10 s at low power using an MSE probe sonicator). A small aliquot of the DPC sample was set aside for DNA quantification and treated with proteinase K (20 mg/ml, Fisher Scientific, BP1700-100), and the remaining DNA was quantified using PicoGreen according to the manufacturer’s instructions (Invitrogen, P7581). After the DNA quantification of each sample, all DPC isolates were normalized to the same amount of DNA and treated with benzonase nuclease (Millipore, E1014) for 1 h at 37°C, followed by snap-freezing in liquid nitrogen and subsequent overnight lyophilization: freeze-drying at −50°C at 5 Pa vacuum using a FreeZone 2.5 lyophilizer (Labconco, USA). Lyophilized DPC samples were dissolved in SDS loading buffer [4 M urea, 62.5 mM Tris–HCl (pH 6.8), 1 mM EDTA, 2% SDS].

To detect total DPCs, dissolved DPC samples were resolved on SDS–PAGE gradient gel (5%–18%) and visualized by silver staining, according to the manufacturer’s protocol (Sigma–Aldrich, PROTSIL1). Specific DPCs were detected by western blot (for histone H3) or by dot blot (for Dnmt1, Top2, Top1, Parp1, Polr3a, and Mcm2) with protein-specific primary antibodies. Briefly, for western blot, DPCs were resolved on 5%–18% SDS–PAGE gradient gels and transferred to PVDF membranes (Roche, 03010040 001). Membranes were blocked with 5% low-fat milk in TBST buffer [20 mM Tris–HCl (pH 7.5), 150 mM NaCl, 0.1% Tween 20] for 2 h at RT and then incubated with primary antibody in 2.5% BSA in TBST overnight at 4°C, followed by incubation with an HRP-conjugated anti-rabbit secondary antibody (Sigma–Aldrich, #A0545, 1:100 000) for 1 h and washing three times for 10 min in TBST buffer. Detection was performed according to the manufacturer’s instructions for the ECL blotting substrate (Bio-Rad, #1705061) and visualized using the ChemiDoc™ XRS + System (Bio-Rad, #1 708 299). For the detection of histone H3, a DPC equivalent of 50 ng total DNA was subjected to western blot and immunostained with the anti-histone H3 antibody (Cell Signaling Technology, #9715, 1:3000). Other specific DPCs (Dnmt1, Top2, Top1, Parp1, Polr3a, and Mcm2) were detected by dot blot with protein-specific antibodies using the Bio-dot microfiltration device (Bio-Rad Laboratories, CA, USA). Briefly, for dot blot, 200 μl of DPC sample diluted in TBST buffer per dot was loaded onto the nitrocellulose membranes (GE Healthcare, 10-6000-02), and the samples were vacuumed using vacuum pump at 700 mbar. For the detection of Dnmt1, Top2, Top1, Parp1, Polr3a, and Mcm2, a DPC equivalent of 25 ng total DNA was subjected to dot blot and immunostained with the following primary antibodies respectively: anti-DNMT1 (Cell Signaling Technology, #5032, 1:1000), anti-TOP2A (Abcam, ab52934, 1:1000), anti-TOP1 (Santa Cruz, sc-271285, 1:1000), anti-PARP1 (Cell Signaling Technology, #9532, 1:1000), anti-POLR3A (Cell Signaling Technology, #12825, 1:1000), and anti-MCM2 (Cell Signaling Technology, #3619, 1:1000). Other steps were performed as previously described for western blot. DNA detection was performed by applying 2 ng of DNA from each sample treated with proteinase K to a nylon membrane and immunostaining with the anti-dsDNA antibody (Abcam, #ab27156, 1:9000) using the dot plot apparatus as previously described. HRP-conjugated anti-rabbit secondary antibody was used for anti-DNMT1, anti-TOP2A, anti-PARP1, anti-POLR3A, and anti-MCM2 primary antibodies, while HRP-conjugated anti-mouse secondary antibody (Sigma–Aldrich, #A9044, 1:100 000) was used for anti-TOP1 and anti-dsDNA primary antibodies.

### Data quantification and statistical analysis

Quantification of silver-stained gels, dot blot and western blot images was performed using ImageJ [54]. GraphPad Prism software (v.9.0.0, GraphPad) was used to generate graphical representations and to statistically analyze the data. Excel (Microsoft) was used to represent the phenotype categories (Categories #1–5), as a percentage of the total number of embryos. Image quantifications and embryo survival rates are shown as mean (SD), while relative gene expression was shown as mean [standard error of the mean (SEM)]. Depending on the experimental conditions, unpaired two-tailed Student’s *t*-test or one-way Analysis of Variance (ANOVA) analyses were performed; differences between two conditions were considered statistical significance when *P* <.05 (ns = not significant for *P* >.05; *P ≤.05; **P ≤.01; ***P ≤.001; ****P ≤.0001).

## Results

### ACRC/GCNA is a highly conserved protein with a SprT-like domain

**Figure 1.**
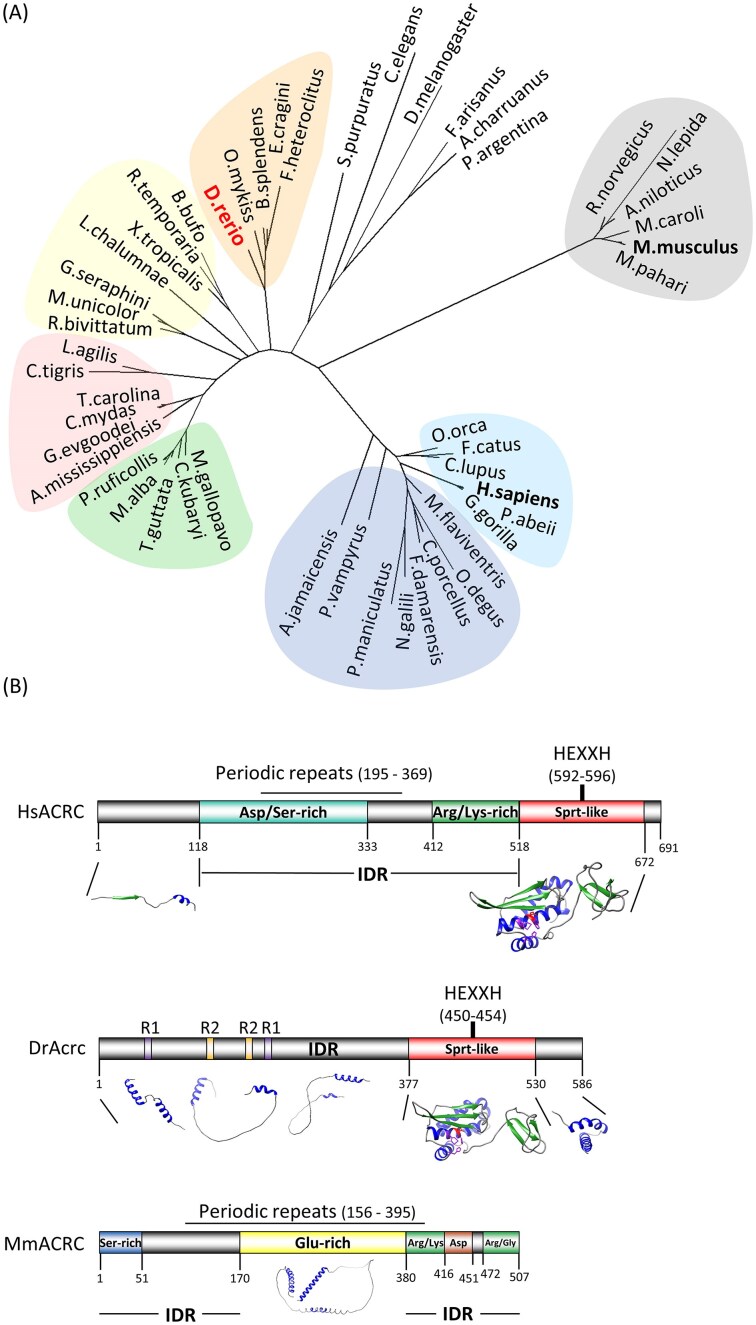
ACRC is an evolutionarily conserved putative protease. **(A)** Phylogenetic analysis of ACRC/GCNA orthologs. Full-length (LG 4) protein sequences were aligned using MAFFT and the phylogenetic tree was constructed in PhyML. Blue: nonrodent mammals; dark blue: rodents and bats with a SprT-like domain; grey: rodents without SprT-like domain; green: birds; red: reptiles; yellow: amphibians; orange: fish; colourless: invertebrates. **(B)** Comparison of human, zebrafish, and mouse ACRC/GCNA protein domains and motifs and structural models of human and zebrafish ACRC. Domains and motifs were identified using SAPS and Motif Scan workspace. Models were built using Alphafold and analyzed with Chimera. HEXXH, protease core; R, repeats; IDR, intrinsically disordered region. The metalloprotease core of ACRC includes the active glutamate shown in red and Zn^2+^-binding histidines shown in orange.

The ACRC protein is highly conserved with one-to-one orthology throughout the animal kingdom (Fig. 1A and Supplementary Fig. S1–S3A). Except for a subset of rodents that includes the house mouse (*Mus musculus*) and the rat (*Rattus norvegicus*), all vertebrate and invertebrate orthologs have a SprT-like domain (Fig. 1A and B, and Supplementary Fig. S2). When we performed a phylogenetic analysis using the full-length protein sequences, the rodent subgroup lacking the SprT-like domain clustered separately from other mammalian orthologs including humans and other rodents (Fig. 1A). As expected, when we used the protein alignment of ACRC SprT-like domains, the phylogenetic tree was very similar to the analysis when the complete protein sequences were used (Supplementary Figs S1 and S2). However, when we performed a phylogenetic analysis using the N-terminal domains and excluding SprT-like domains, all mammalian orthologs clustered together, indicating that the rodent subgroup is evolutionarily close to other mammalian groups with a SprT-like domain (Supplementary Fig. S3A and Supplementary Table S1).

As the SprT-like domain is a likely active protease domain which could have a function in DPCR, we specifically analyzed the evolutionary conservation and protein structure of this domain. The 3D Alphafold structural model shows a high degree of conservation between the human and zebrafish SprT-like domains. Both domains were modelled with very high or high degree of confidence (per-residue confidence score, pLDDT >90 and 70–90, respectively) (Fig. 1B). The ACRC protease core comprises three α-helices and three β-sheets carrying the active site, which consists of a catalytic glutamate (E593 in human and E451 in zebrafish) and three Zn^2+^-binding histidines (H592, H596, and H609 in human and H450, H454 and H467 in zebrafish) (Fig. 1B). Three β-sheets are conserved at the C-terminal end of the SprT-like domain in both species. Both proteins also share a long unstructured and intrinsically disordered region (IDR ) in the N-terminal part (Fig. 1B and Supplementary Fig. S3B). Interestingly, the human and zebrafish orthologs differ considerably in their protein charge clusters in the IDR region: human ACRC carries a large negative (Asp/Ser-rich) and a large positive charge cluster (Lys/Arg-rich), whereas zebrafish Acrc does not have significantly charged regions within the IDR (Fig. 1B). In addition, the nature of the amino acid repeats within the IDR differs: human ACRC contains many periodic repeats spanning a large portion of the IDR, whereas zebrafish Acrc has only two short repeats (R1 and R2) (Fig. 1B). The Alphafold model of the C-terminal part of zebrafish Acrc predicts two α-helices with high to very high confidence, as well as intermittent structured regions (low to high confidence) within the otherwise largely disordered N-terminal part (Fig. 1B). In comparison, the human ACRC has a shorter, unstructured C-terminus and is largely disordered in the N-terminal part (Fig. 1B and Supplementary Fig. S3B). Interestingly, when we compared the human and zebrafish protein regions outside the SprT-like domain with their mouse ortholog, we observed similar large unstructured and disordered regions, including periodic repeats, whereas the charge clusters of mouse Acrc are more similar to its human ortholog (Fig. 1B).

Human and mouse *ACRC* genes are located on the X chromosome, whereas zebrafish *acrc* is positioned on chromosome 14 (Supplementary Fig. S4). Of note, zebrafish do not have sex chromosomes, but one of the chromosome clusters that determine gender is located on chromosome 14 [55]. Otherwise, the gene environments of the human and mouse *ACRC* orthologs are quite similar and partially conserved with the gene environment of zebrafish *acrc* (Supplementary Fig. S4).

In summary, phylogenetic, syntenic and domain analyses showed that zebrafish is a good model to study ACRC function because it shares many features with human ACRC, including the SprT-like domain that the mouse ortholog lacks.

### *Acrc* and *sprtn* are highly expressed during embryonic development and in adult tissues

**Figure 2.**
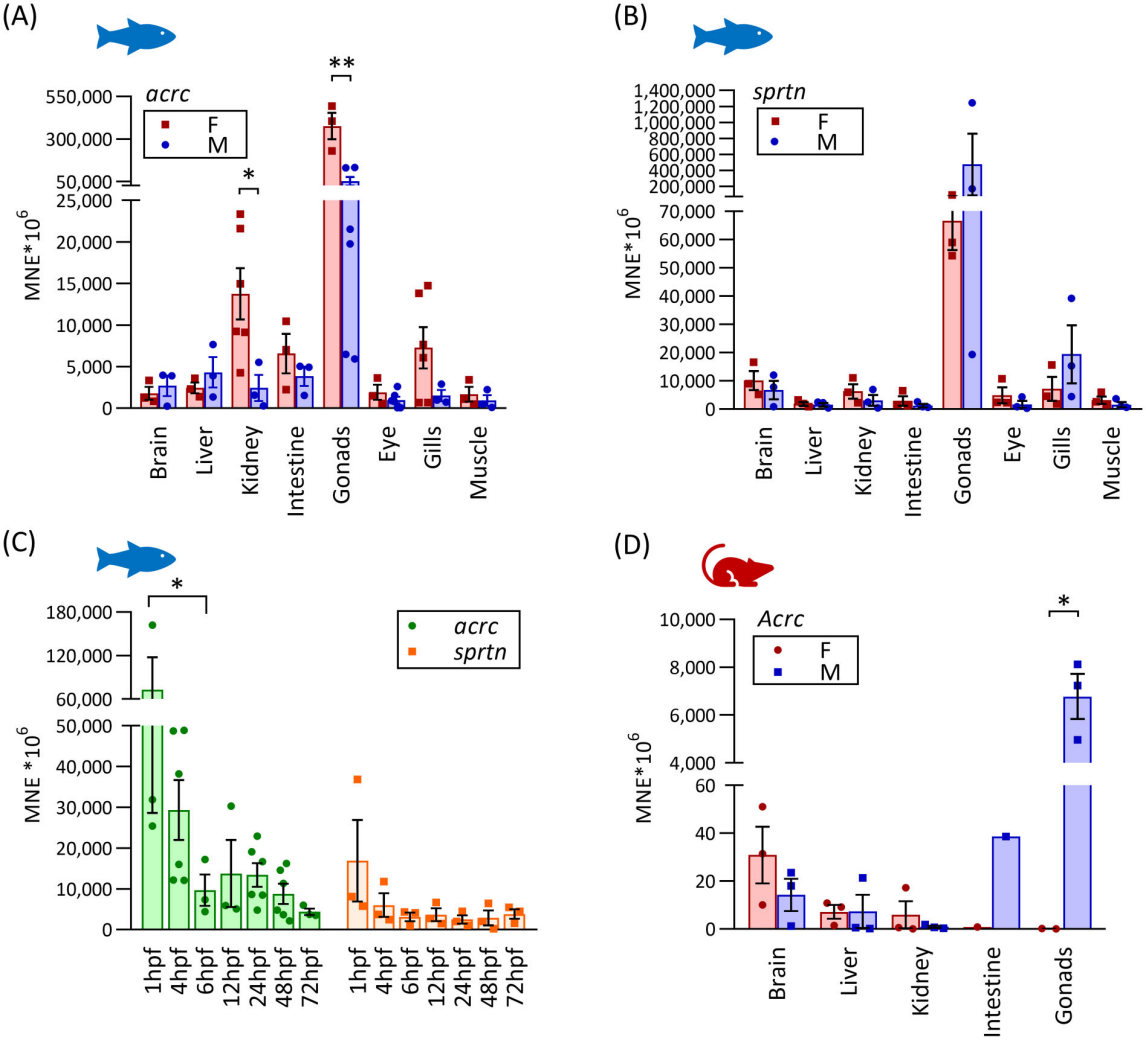
Gene expression of *acrc*  **(A)** and **(B)**  *sprtn* in tissues of adult female (F) and male (M) zebrafish. Data represent MNE from individual experiments (±SEM), normalized to the reference gene *elongation factor 1α (eef1a1l1*). Only statistically significant differences are shown on the graph (unpaired Student’s *t*-test, *P <.05; **P <.005). **(C)** Gene expression of *acrc* and *sprtn* in zebrafish embryos during development (hpf, hours post fertilization). Data represent MNE from individual experiments and (±SEM), normalized to the reference gene *eef1a1l1*. Only statistically significant differences are shown on the graph (one-way ANOVA, Sidak’s multiple comparisons test *P <.05). **(D)** Gene expression of *Acrc* in tissues of adult mice. Data represent MNE from individual experiments (±SEM), normalized to the reference gene *Rplp0*. Only statistically significant differences are shown on the graph (unpaired Student’s *t*-test, *P <.05).

We determined the mRNA expression of *acrc* in adults and embryos and compared it with the expression of *sprtn* to gain more insight into these two DPC proteases. To facilitate comparison of expression levels, we set arbitrary thresholds following previous publications [52, 56, 57]: high expression when MNE is >60 × 10^6^, moderate when MNE is 2 × 10^6^–60 × 10^6^ and low when MNE is <2 × 10^6^. *Acrc* shows very high expression in the gonads, particularly in the ovaries, where it is expressed 5.6-fold more than *sprtn* (Fig. 2A and B, and Supplementary Table S2A and B). In comparison, the expression of *sprtn* in the testes is very high: nine-fold higher than that of *acrc*. In all other tissues examined, including brain, liver, kidney, intestine and gill, we found moderate to high expression of both genes (Fig. 2A and B, and Supplementary Table S2A and B).

Next, we determined the expression levels of *acrc* and *sprtn* during the embryonic development when replication and transcription rates are very high and accumulated DPCs can be especially detrimental. Gene expression levels of *acrc* and *sprtn* are high in zebrafish embryos between 1 and 72 hpf (Fig. 2C and Supplementary Table S2C). Both genes showed the highest expression at 1 hpf, indicating maternal mRNA deposition in the egg, as zygotic transcription is only fully activated at 3 hpf and maternal transcript levels decrease between 2 and 3 hpf [58, 59]. Expression levels of *acrc* decline rapidly until 6 hpf and remain approximately stable until 48 hpf before decreasing further. In comparison, expression levels of *sprtn* also decrease rapidly until 6 hpf, but remain stable until 72 hpf. *Acrc* levels were at least three times higher than *sprtn* levels at all stages until 48 hpf, indicating a crucial role during development. It is important to note that the expression levels of both genes are still high at 72 hpf (MNE 3809–4853 × 10^6^) (Fig. 2C and Supplementary Table S2C).

To further investigate the role of the SprT-like-domain, we compared the gene expression levels of zebrafish *acrc* (which has a SprT-domain) and mouse *Acrc* (which lacks the SprT domain) in adult tissues. The expression pattern differs markedly between the two genes, with zebrafish *acrc* being moderately to highly expressed in all examined tissues, with the highest expression in ovary and testis, whereas mouse *Acrc* is dominantly expressed in testis, and at low to moderate levels in other tissues (Fig. 2A and D, and Supplementary Tables S2A and D).

### Acrc mutations in the SprT-like domain cause early embryonic lethality in zebrafish

**Figure 3.**
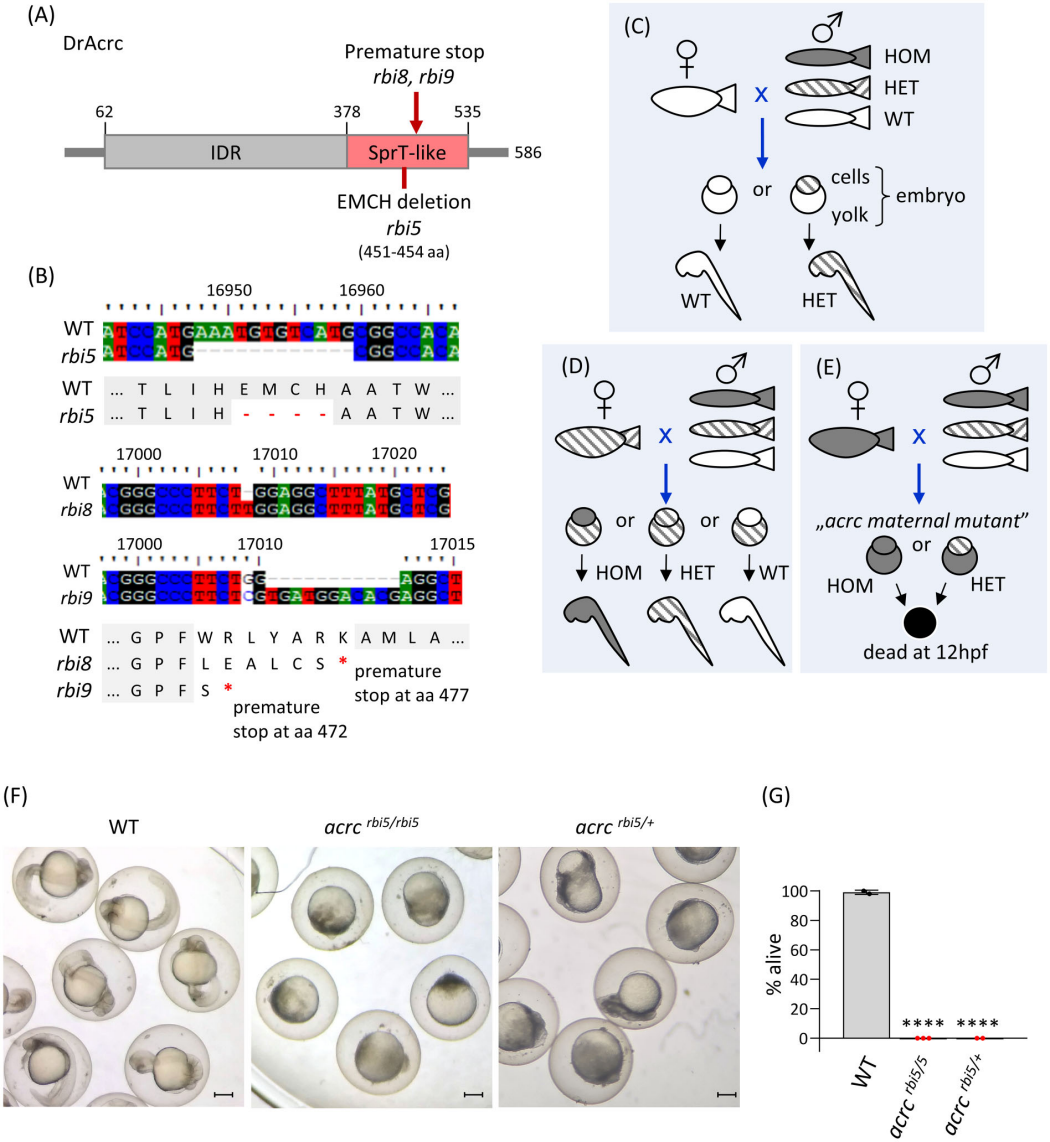
Creation of *acrc* mutant zebrafish strains. **(A, B)** Scheme of the Acrc protein showing the sequences and positions of the 4 amino-acids deletion (ΔEMCH, 451–454 aa) in the enzymatic core of the SprT-like domain in the *rbi5* allele, and of the premature stop codons resulting from the *rbi8* and *rbi9* alleles **(C–E)** Schemes representing genotype-phenotype correlations upon loss of one or both *acrc* wild type (wt) alleles: **(C)** crossing a WT female with any male (WT: white, heterozygote: stripes, or mutant: grey) always leads to viable fish; **(D)** crossing a heterozygous female with any male (WT, heterozygote or mutant) always leads to viable fish; and **(E)** crossing an *acrc* mutant female with any male (WT, heterozygote or mutant) always results in embryos with an early lethal phenotype (black yolk). **(F)** Representative images of WT, mutant and heterozygous (from homozygous females crossed with WT males) zebrafish embryos at 24 hpf. Scale bars: 250 µm. **(G)** Quantification of embryo survival at 24 hpf. Data are shown as the percentage of live embryos from at least two independent experiments with at least 15 embryos each (mean ± SD; one-way ANOVA, ****P <.0001).

To study DPC repair *in vivo*, we generated two zebrafish *acrc* mutant strains targeting the SprT-like domain using the CRISPR/Cas9 system. One mutant strain carries an in-frame deletion of 12 nucleotides in the putative catalytic site, resulting in the deletion of the catalytic glutamate E451 and the following three amino acids in the protease core, including the Zn-bearing histidine H454 (ΔEMCH) (Fig. 3A and B). We named this mutant allele *rbi5* (*ruder boskovic institute* 5) following the nomenclature suggested by ZFIN (The Zebrafish Information Network, zfin.org). The second mutant strain carries mutations in exon 12, leading to frameshift and premature stop codons at amino acid positions 477 and 472 (Fig. 3A and B). We named these mutant alleles *rbi8* and *rbi9* (*ruder boskovic institute* 8 and 9).

The Acrc protein is translated in both mutant strains: *acrc^rbi5/rbi5^* (ΔEMCH) and *acrc^rbi8/rbi9^*, and protein levels are similar to those in WT fish (Supplementary Fig. S5A). It is important to note that the DrAcrc protein migrates on SDS–PAGE at an apparent MW of 100 kDa, instead of its true size of 65 kDa due to its very high hydrophobicity, intrinsic disorder, and large hydrodynamic radius, clusters of acidic residues and high proline content. These properties of intrinsically disordered proteins typically result in 1.2–1.8 times slower migration through the gel because of reduced SDS binding and a more extended protein conformation, a phenomenon known as gel shifting [60–62]. As expected, the truncated Acrc protein (MW 53 kDa) in the mutant strain *acrc^rbi8/rbi9^* migrates slightly faster than WT on an SDS–PAGE gel (Supplementary Fig. S5A).

Deletion of EMCH in the SprT-like domain of zebrafish Acrc does not affect the model of 3D protein structure (very high confidence; pLDDT > 90) and the two main α-helices within the putative protease core remain intact (Supplementary Fig. S5B). EMCH residues form one turn in one of the helices in the WT, whereas in the mutant, this turn is ‘’skipped’’ and the helix is shorter for it (Supplementary Fig. S5B). In addition, in order to assess the thermodynamic impact of the EMCH deletion, we compared the energy parameters between Acrc-WT and Acrc-ΔEMCH using FoldX [63]. The deletion of the EMCH residues from an α-helix in the protease domain results in a 17.77 kcal/mol destabilization (ΔΔG = 111.40 − 93.63 = +17.77 kcal/mol) which is thermodynamically significant (Supplementary Fig. S5C). However, when comparing the individual energy parameters some changes indicate stabilizing effects, while others are destabilizing (Supplementary Fig. S5C). For example, the dipole moment slightly improved in the mutant, which probably explains why the shorter helix is predicted to fold normally (with high confidence). In conclusion, considering that the experimental data shows normal protein levels and correct molecular weight (Supplementary Fig. S5A), while the structural models from Alphafold predict normal folding, we conclude that the deletion does not cause protein degradation and it probably does not cause protein misfolding *in vivo*.

In the *acrc^rbi8/rbi9^* mutants, the premature stop occurs shortly after the third Zn-bearing histidine (H467) (Fig. 3B), resulting in alterations to the Sprt domain (Fig. 3A). The truncated Acrc protein is translated and its levels are similar to those of Acrc in WT fish (Supplementary Fig. S5A). The 3D structural model shows that one α-helix forms, as in WT (very high confidence; pLDDT > 90), while the other α-helix containing the third Zn-bearing histidine (H467) does not form, due to the premature stop. Surprisingly, energy parameters of truncated Acrc indicate that truncation leads to overall stabilization of the protein (ΔΔG = 76.51 − 93.63 = −17.12 kcal/mol) (Supplementary Fig. S5C). This counterintuitive stabilization of the truncated form arises from competing thermodynamic contributions shown in Supplementary Fig. S5C that ultimately favor the mutant. Overall, based on *in silico* analysis, western blot, and functional data, we conclude that truncated Acrc is not degraded in mutant embryos. However, it is functionally inactive: although truncated Acrc retains the catalytic glutamate and three Zn-bearing histidines, the second α-helix carrying H467 does not fold (Supplementary Fig. S5B), and part of the Sprt domain (residues 472–535) is missing in the mutant (Fig. 3A and B).

The phenotype of both types of mutant strains is maternal zygotic embryonic lethal, with lethality occurring prior to 24 hpf (Fig. 3C–G and Supplementary Figs S6–S8A). Most importantly, the fact that the catalytic mutation (rbi5, ΔEMCH) in the protease core causes embryonic lethality suggests that it is the proteolytic function of Acrc that is responsible for the observed phenotype. The maternal zygotic phenotype is apparent from the fact that heterozygous embryos derived from WT fathers and homozygous mutant mothers show an early lethality phenotype which could be rescued by injecting one-cell stage embryos with *in vitro* synthesized mRNA encoding Acrc-WT (Fig. 3F and G, and Supplementary Table S3A). In contrast, homozygous mutant embryos derived from heterozygous parents show no morphological defects (Fig. 3C–E and Supplementary Fig. S8A) which demonstrates the requirement for maternal deposition of functional Acrc protein and/or mRNA in the eggs for normal embryonic development.

### The putative catalytic core of the SprT-like domain is essential for Acrc function during early embryonic development

**Figure 4.**
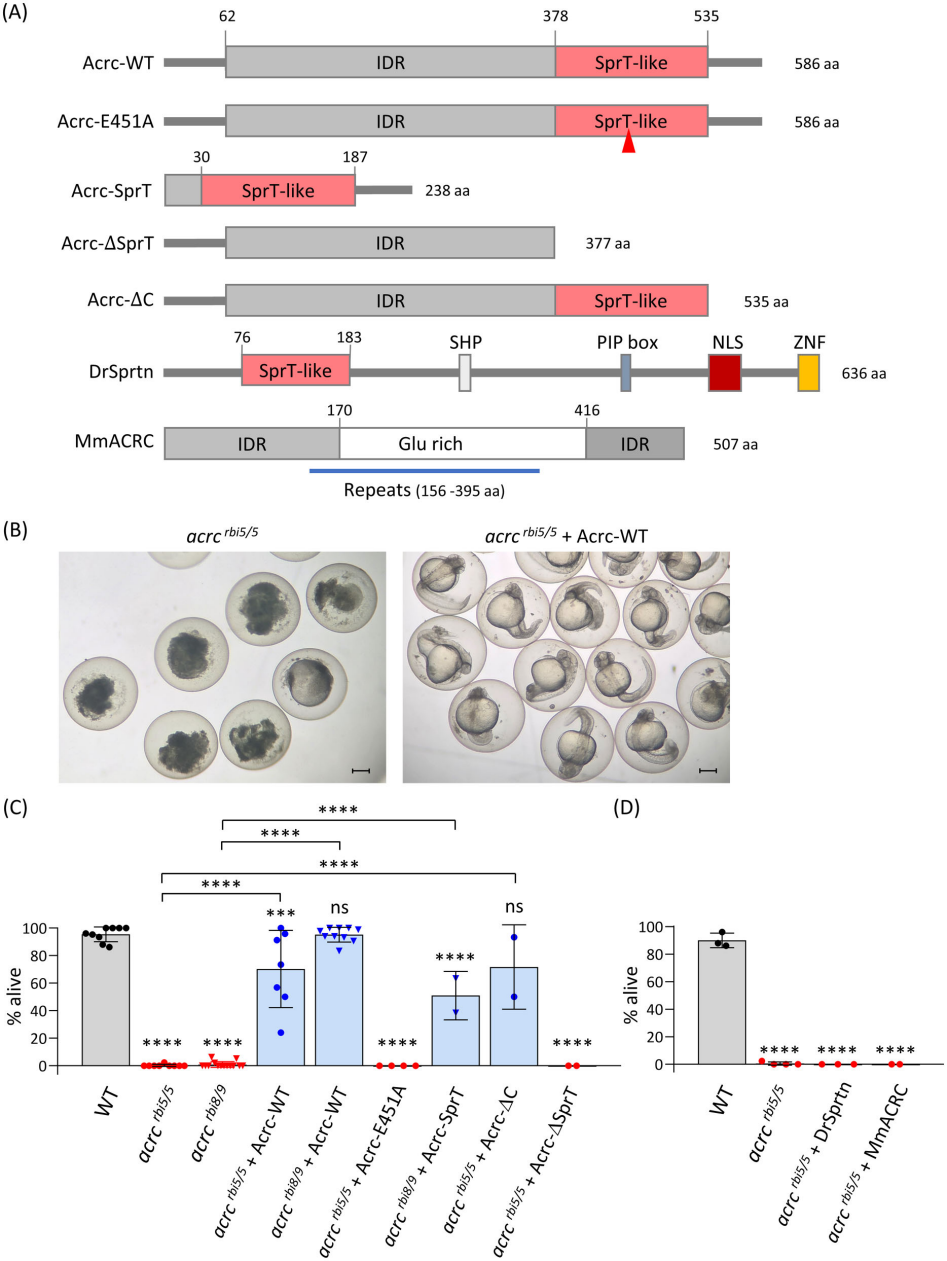
Injection of Acrc-WT mRNA rescues the *acrc* mutant phenotype, while injection of Acrc-E451A, DrSprtn, and MmAcrc mRNA does not. **(A)** Schemes of the injected rescue constructs: Acrc-WT, Acrc-E451A, Acrc-SprT, Acrc-ΔSprT (IDR), Acrc-ΔC, and DrSprtn and MmACRC; Mm, *Mus musculus*; Dr, *Danio rerio*. **(B)** Representative pictures of 24 hpf *acrc^rbi5/rbi5^* mutant embryos and *acrc^rbi5/rbi5^* mutant embryos injected with Acrc-WT mRNA at the one-cell stage. Scale bars: 250 µm; **(C, D)** Quantification of *acrc* mutant embryo survival at 24 hpf after injection with the rescue constructs shown in panel (A). Data are presented as the percentage of live embryos from at least two independent experiments with at least 15 embryos each (mean ± SD). Each condition was compared to WT (stars and “ns” above each column), and conditions with rescued embryos were compared to the respective uninjected *acrc^rbi5/rbi5^* or *acrc^rbi8/rbi9^* mutants; one-way ANOVA and Dunnett’s test (****P-value <.0001; ***P-value *P* <.001; ns: not significant).

To confirm that the catalytic mutation is responsible for the lethal phenotype and to gain insight into the function of other Acrc domains, we injected *in vitro* transcribed and capped mRNA encoding either WT or mutated versions of Acrc (Fig. 4A) into *acrc^rbi5/rbi5^* mutant embryos at the one-cell stage and examined whether they could compensate for the loss of Acrc and rescue the early lethality phenotype of the *acrc* mutants.

Each *in vitro* synthesized mRNA was injected into WT and *acrc* mutant embryos, and the resulting phenotype of each embryo was scored after 24 hpf according to severity in five categories: WT-like (alive), abnormal (alive), very abnormal (alive), necrotic (dead), and dead (Supplementary Figs S6A and B, and S8B and D). To statistically compare the rescue efficiency of the various constructs, we grouped the phenotypes into a binary system: “alive” or “dead” (Fig. 4C, and Supplementary Fig. S6C, and S8C and E). This was performed in both, *acrc^rbi5/rbi5^* and *acrc^rbi8/rbi9^* mutants, as well as in WT embryos as a control (Fig. 4, Supplementary Figs S6 and S8, and Supplementary Table S3).

Firstly, we injected an Acrc-WT mRNA into mutant embryos and observed a rescue of the lethal phenotype (Fig. 4B and C, and Supplementary Fig. S6C). After 24 hpf, 70.2% of *acrc^rbi5/rbi5^* mutant embryos and 95.1% of *acrc^rbi8/rbi9^* mutants injected with Acrc-WT mRNA were alive (Supplementary Table S3B). The degree of rescue was such that in fact, the majority of injected embryos (>50%) developed normally to adulthood (3 months old) and older age (1.5 years old). An example of uninjected and injected *acrc^rbi8/rbi9^* maternal mutants at 24 hpf can be seen in Supplementary Fig. S7.

Secondly, we showed that the catalytic glutamate E451 in the putative protease core is essential for embryonic development, as the injected Acrc-E451A mRNA could not rescue the lethal phenotype (Fig. 4C, Supplementary S6 and S7, and Supplementary Tables S3B and C). After 24 hpf, none of the mutant embryos (*acrc^rbi5/rbi5^* and *acrc^rbi8/rbi9^)* injected with Acrc-E451A mRNA were alive (Fig. 4C, Supplementary Fig. S6, and Supplementary Tables S3B and C), suggesting that the glutamate 451 located in the protease core is crucial for Acrc function during early embryonic development. The presence of Acrc-E451A mRNA was confirmed using qPCR (Supplementary Fig. S9A), while the protein translation was confirmed using anti-myc immunostaining (Supplementary Fig. S9B).

Next, we tested whether the other domains of Acrc affect the lethality phenotype. To this end we tested the function of a SprT domain (Acrc-SprT), IDR domain (Acrc lacking the SprT-like domain named Acrc-ΔSprT), and a truncated version of Acrc lacking the C-terminal tail (Acrc-ΔC).

Acrc-SprT, a truncated version of Acrc lacking the entire IDR domain and consisting mostly of the SprT-like domain, was injected into *acrc^rbi8/rbi9^* mutants (Fig. 4C and Supplementary Fig. S6B). This construct significantly rescued the lethality phenotype, with 50.9 ± 17.6% of live embryos after 24 hpf (Fig. 4C and Supplementary Fig. S6B, and Supplementary Table S3B). The rescue was not as strong as with the Acrc-WT construct (95.1 ± 5.4%) (Fig. 4C and Supplementary Table S3B). The Acrc construct consisting mostly of the IDR domain (Acrc-ΔSprT) was not able to rescue the lethal phenotype (Fig. 4C, Supplementary Fig. S6, and Supplementary Tables S3B and C). The construct with small C-terminal truncation, which has intact SprT and IDR domains (Acrc-ΔC), was able to rescue the lethal phenotype, similar to Acrc-WT (Fig. 4C, Supplementary Fig. S6, and Supplementary Tables S3B and C): 71.7 ± 30.6% of *acrc^rbi5/rbi5^* maternal mutant embryos injected with Acrc-ΔC mRNA were alive after 24 hpf, while 93.6 ± 9.9% *acrc^rbi8/rbi9^* maternal mutants were alive after injection with the Acrc-ΔC mRNA (Fig. 4C, Supplementary Fig. S6, and Supplementary Tables S3B and C). Overview pictures of injected embryos from representative rescue experiments are shown in Supplementary Fig. S7.

Protein translation of all injected mRNAs was confirmed by anti-myc immunostaining of 6 hpf embryos (Supplementary Fig. S9B), since all the translated proteins have a myc tag translated in frame at the N-terminus. Finally, we verified that expression of the rescue constructs did not impair embryo development with control injections into WT embryos (Supplementary Fig. S8 and Supplementary Tables S3D and E).

Overall, the rescue experiments demonstrate that the intact protease core of Acrc is essential for survival at early embryonic stages. Remarkably, *acrc* mutant embryos of both strains (*rbi5/rbi5* and *rbi8/rbi9*) injected with full-length Acrc-WT mRNA could reach adulthood and were fertile, allowing us to breed and maintain the *acrc* mutant lines.

### Mouse and human ACRC did not compensate for loss of zebrafish *acrc* during early embryonic development

To further confirm that the proteolytic function of ACRC is essential for the early development, we tested whether mouse Acrc, which lacks a SprT-like domain (Fig. 1B), can rescue early embryonic lethality in *acrc* mutant fish (*acrc^rbi5/rbi5^*). To this end, we injected mouse Acrc mRNA into zebrafish mutant embryos (*acrc^rbi5/rbi5^* and *acrc^rbi8/rbi9^*) (Fig. 4A and D, Supplementary Figs S6B and C, and S10A and E, and Supplementary Tables S3C and F). None of the injected embryos were alive at 24 hpf (Fig. 4D, Supplementary Fig. S6B and C, and S10A and E, and Supplementary Table S3C and F). Control injections into WT confirmed that injecting mouse Acrc mRNA is not toxic for zebrafish embryos (Supplementary Fig. S10B and C, and Supplementary Table S3G). After 24 h, 91% of WT embryos injected with mouse Acrc mRNA were alive, but we observed higher number of abnormal embryos in comparison to WT (Supplementary Fig. S10B and Supplementary Table S3G). In addition, PCR confirmed that mouse Acrc mRNA remains stable for at least 2 days after the injection (Supplementary Fig. S10D), while immunostaining confirmed that mouse Acrc protein is translated and located in the nuclei and the DAPI-stained nuclei of mutants after the overexpression of mouse Acrc appeared visually normal (Supplementary Fig. S9B).

In contrast, the injection of human ACRC mRNA (Supplementary Fig. S10F) into *acrc^rbi8/rbi9^* mutant embryos caused defects in the nuclei of 6 hpf embryos. DAPI staining revealed abnormal cell divisions and size of the nuclei (Supplementary Fig. S9B and C). The human ACRC mRNA did not rescue the lethality phenotype (Supplementary Figs S6B and C, and S10E, and Supplementary Table S3C), which could be due to the observed changes in the nuclei. Immunostaining showed that HsACRC was expressed and localized to the nuclei (Supplementary Fig. S9B, right panel). However, human ACRC formed distinct nuclear foci which were not observed with zebrafish, nor mouse Acrc (Supplementary Fig. S9B). The overexpression of human ACRC was not toxic to the WT embryos: 91.6 ± 8.3% of WT injected with HsACRC mRNA were alive after 24 hpf (Supplementary Fig. S8D and E, and Supplementary Table S3E).

### Sprtn did not compensate for loss of zebrafish *acrc* during early embryonic development

Considering that the protease Sprtn, like Acrc, has a SprT-like domain (Fig. 4A) and that they have very similar protease cores [4], we investigated whether zebrafish Sprtn can compensate for the absence of Acrc during early development. At 6 hpf, *sprtn* is highly expressed in zebrafish embryos, in both WT and *acrc* mutants (MNE = 955–4331 × 10^6^) (Fig. 2C, Supplementary Fig. S10G–H, and Supplementary Tables S2C), and this level of expression is not sufficient to compensate for the loss of Acrc function in the mutants. We then tested whether Sprtn overexpression would rescue the lethality phenotype. After the injection of Sprtn mRNA into *acrc^rbi5/rbi5^* mutant embryos, Sprtn failed to rescue the early lethality phenotype as not a single viable embryo was present at 24 hpf (Fig. 4D and Supplementary Fig. S10A, and Supplementary Table S3F). The same result was observed in *acrc^rbi8/rbi9^* mutants (Supplementary Figs S6B and C, and S10E, and Supplementary Table S3C). A control experiment with injection of DrSprtn in WT embryos showed that overexpression did not affect WT viability (Supplementary Fig. S10B and C, and Supplementary Table S3G). In addition, Sprtn mRNA was stably expressed (Supplementary Fig. S9A), and the protein was translated and localized to the nucleus (Supplementary Fig. S9B).

### *Acrc* mutants accumulate DPCs before the onset of lethality

After we showed that intact protease core of Acrc is required for embryonic development, we investigated whether the molecular mechanism behind the embryonic lethality is impaired DNA–protein crosslink repair.

**Figure 5.**
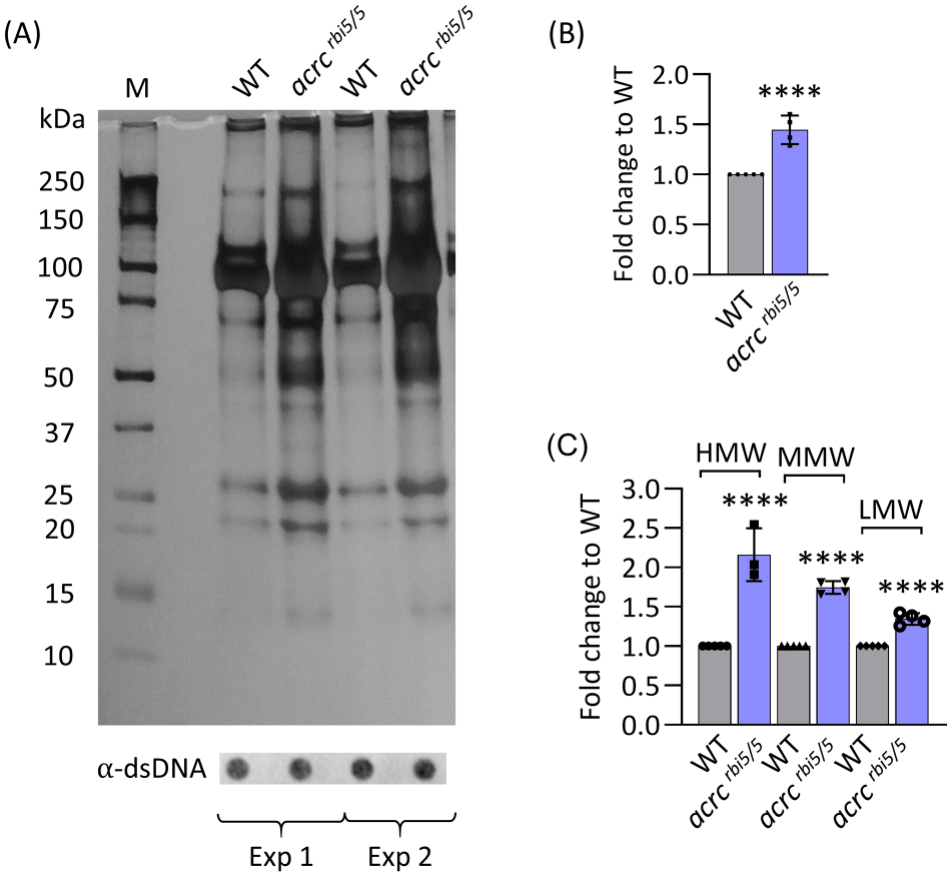
DNA–protein crosslinks accumulate in *acrc* mutant embryos. **(A)** Total cellular DPCs in WT and *acrc* mutant embryos, resolved on SDS–PAGE gels and stained with silver. DPCs were isolated using modified RADAR method from 6 hpf WT and *acrc^rbi5/rbi5^* embryos (*n* = 50, four independent experiments). Dot-blots showing DNA loading controls for DPC analysis prior to benzonase treatment are shown below; a DPC equivalent of 25 ng total DNA was loaded per well (M, molecular weight marker); **(B)** Quantification of panel (A) using ImageJ based on data shown here and in Supplementary Fig. S11A (four biological replicates). **(C)** Quantification of HMW-DPCs (high molecular weight DPCs, Mr > 151 kDa), MMW-DPCs (medium molecular weight DPCs, Mr = 41–150 kDa), and LMW-DPCs (low molecular weight DPCs, Mr < 40 kDa) from panel (A). Data are shown as mean ± SD (*n* = 4) and statistically significant differences compared to WT embryos are indicated (****P <.0001, Student’s *t*-test).

To this end, we isolated DPCs from 6 hpf old mutant embryos before the onset of lethality, when mutants looked indistinguishable from WT embryos. We used the modified RADAR isolation protocol which we previously adapted for zebrafish embryos [52]. *Acrc^rbi5/rbi5^* mutants had significantly increased levels of total cellular DPCs compared with WT embryos (1.44-fold change) (Fig. 5 and S11A). In order to determine which DPCs are most affected by Acrc defficiency, DPCs were divided into three groups: HMW (>151 kDa), MMW (40–150 kDa), and LMW (5–40 kDa). We are aware that this categorization is not ideal, but analysis of DPC size can help us to better understand how Acrc functions in the DPC repair, considering that the function of a particular repair factor can depend on the size of the crosslinked protein. We show that Acrc deficiency leads to the highest increase in HMW-DPCs, followed by MMW-DPCs and the modest accumulation of LMW-DPCs (Fig. 5C). In addition, we confirmed the total DPC increase in the *acrc* mutants using another DPC isolation method, KCl/SDS DPC isolation (Supplementary Fig. S11B). Using the SDS–KCl method, we precipitated protein-bound DNA to separate it from protein-free DNA. The amount of DNA in the SDS pellet is used as a measure of DNA–protein crosslink amount expressed as a percentage of crosslinked DNA [12, 51]. *Acrc* mutants accumulated significantly more DPCs in comparison to WT embryos at 6 hpf: the percentage of crosslinked DNA relative to free DNA in *acrc^rbi8/rbi9^* mutants was 32.75 ± 2.88%, in comparison to 18.04 ± 3.09% in WT embryos (Supplementary Fig. S11B).

### The catalytic mutation in the Acrc protease leads to the accumulation of Dnmt1-, Top1-, Top2-, histone H3-, Parp1-, Polr3a-, and Mcm2-DPCs

For the quantification of specific DPCs, we used the modified RADAR method for DPC isolation, as it enables the isolation of intact crosslinked proteins and thus subsequent identification of the protein species. We investigated whether previously suggested Acrc substrates, DNA (cytosine-5)-methyltransferase 1 (Dnmt1) and topoisomerase 2 (Top2) [23, 24] are crosslinked in *acrc^rbi5/rbi5^* mutant embryos at 6 hpf when embryos exhibited normal WT-like morphology. In addition, considering that histones are the most abundant DPCs composing approx. 80% of all cellular DPCs [64], we tested whether core histones could be DPC substrates of the Acrc using histone H3 as a representative. Next, we tested whether Acrc is involved in the removal of other abundant cellular DPCs previously identified by mass spectrometry analysis in human cells [64]: TOP1 (DNA topoisomerase 1), PARP1 (Poly [ADP-ribose] polymerase 1), POLR3A (DNA-directed RNA polymerase III subunit RPC1), and MCM2 (Minichromosome maintenance complex component 2).

**Figure 6.**
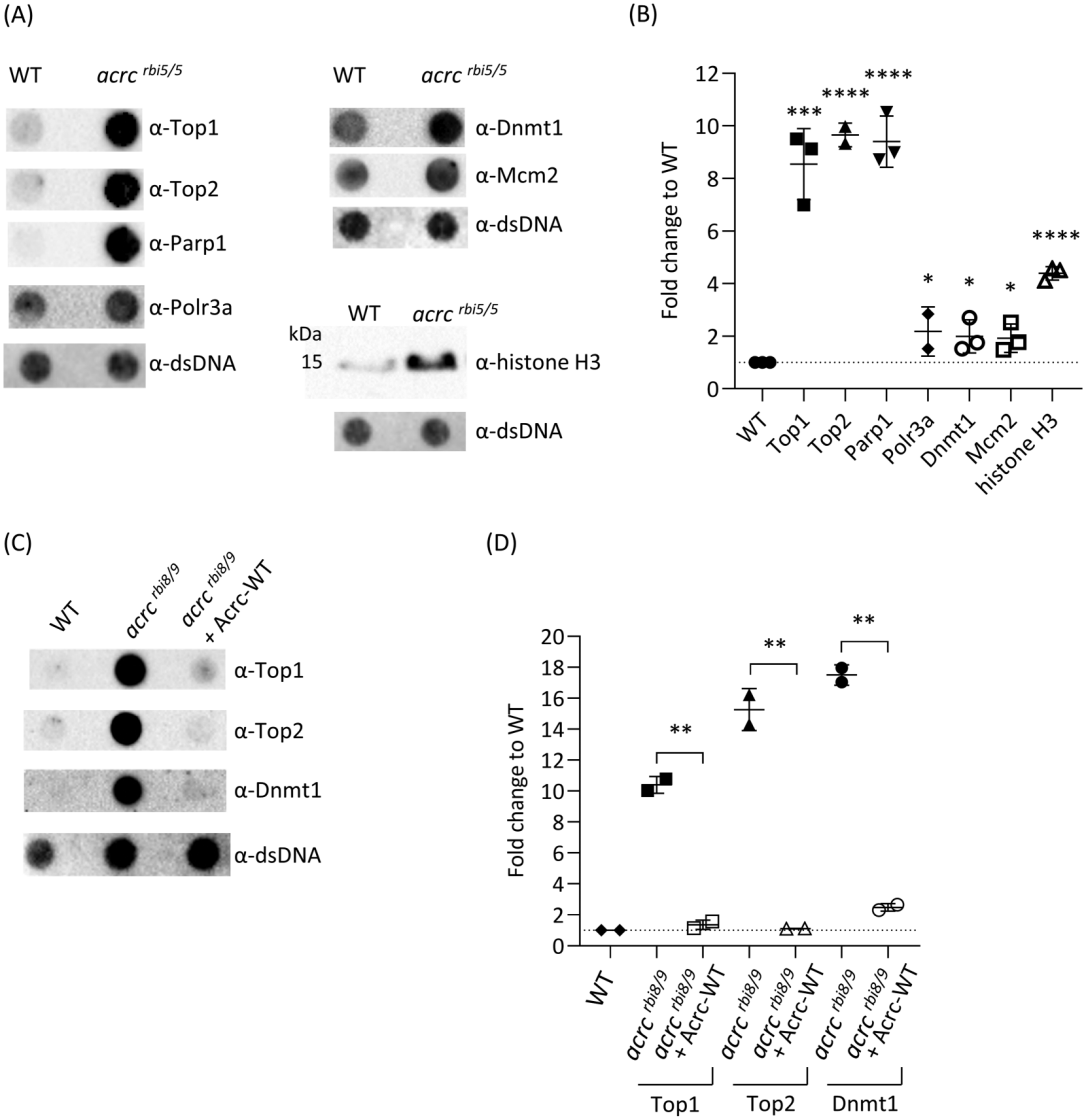
ACRC is involved in the repair of multiple cellular DPCs. **(A)** Top1-, Top2-, Parp1-, Polr3a-, Dnmt1-, Mcm2-, and histone H3-DPCs accumulate in *acrc^rbi5/rbi5^* mutant embryos. DPCs were isolated by RADAR assays from 50 embryos (6 hpf) and visualized using dot blot or western blot with specific antibodies. A DPC equivalent of 25 ng total DNA was loaded per dot. Dot blots showing DNA loading controls for DPC analysis prior to benzonase treatment are shown (2 ng of total DNA were loaded per well); **(B)** Quantification of panel (A) using ImageJ based on data shown here and in Supplementary Fig. S11C (biological replicates). Data are shown as mean ± SD (*n* = 3) and statistically significant differences compared to WT embryos are indicated (*P <.05, ***P <.001, ****P <.0001, Student’s *t*-test). **(C)** Acrc-WT complementation reduces DPC levels in *acrc* mutants. Top1-, Top2-, and Dnmt1-DPC levels in *acrc* mutants are similar to WT embryos after injection of Acrc-WT mRNA in *acrc^rbi8/rbi9^* mutants. DPCs were isolated using a modified RADAR assay from 50 embryos (6 hpf) and visualized using dot blot with specific antibodies. A DPC equivalent of 25 ng total DNA was loaded per dot. Dot blots showing DNA loading controls for DPC analysis prior to benzonase treatment are shown (2 ng of total DNA were loaded per well). **(D)** Quantification of panel (C) using ImageJ, based on data shown here and in Supplementary Fig. S11D. Data are shown as mean ± SD (*n* = 2), and statistically significant differences are indicated (**P <.01, ***P <.001, Student’s *t*-test).

In the absence of functional Acrc, we observed a marked accumulation of Top1-DPCs (8.5 ± 1.35 fold), Top2-DPCs (9.7 ± 0.45 fold), and Parp1-DPCs (9.4 ± 0.98 fold) compared to WT embryos (Fig. 6A and B, and Supplementary Fig. S11A). Histone H3-DPCs were also significantly increased, with a 4.4 ± 0.26 fold change compared to WT (Fig. 6A and B, and Supplementary Fig. S11C). Additionally, we observed a significant increase in Polr3a-DPCs (2.2 ± 0.93 fold), Dnmt1-DPCs (2.0 ± 0.63 fold) and Mcm2-DPCs (1.9 ± 0.54 fold) in the *acrc^rbi5/rbi5^* mutants (Fig. 6A and B, and Supplementary Fig. S11C). To test the specificity of these results, which indicate that Acrc is involved in the repair of multiple cellular DPCs, we isolated and quantified DPCs after injecting Acrc-WT mRNA into *acrc* mutants. WT complementation reduced DPC levels of Top1, Top2, and Dnmt1 to the WT levels (Fig. 6C and D, and Supplementary Fig. S11D).

## Discussion

We report that the Acrc protein is essential during vertebrate embryonic development through its function as a putative DPC repair protease. A functional SprT-like domain, and particularly a catalytic glutamate, are indispensable for the full functionality of Acrc and thus for embryo survival. Our *in vivo* analysis of different Acrc domains showed that intact SprT-like domain is required to rescue the lethality phenotype in *acrc* mutants, while the IDR alone is not sufficient. We further showed that Acrc is involved in the repair of multiple cellular DPCs including Dnmt1, topoisomerases 1 and 2, histone H3, Parp1, Polr3a, and Mcm2. To our knowledge, the findings presented here are novel.

Given its essential function during vertebrate embryonic development, it is not surprising that ACRC is highly conserved and exhibits one-to-one orthology throughout the animal kingdom (Fig. 1 and Supplementary Fig. S1–S3). In support of our functional studies, we found that the SprT-like domain in the C-terminal half of ACRC is remarkably conserved from invertebrates to human (Fig. 1 and Supplementary Fig. S2), while the N-half of the protein is largely disordered and variable across species (Fig. 1A and Supplementary Fig. S3). Considering that the mouse Acrc lacks a SprT-like domain [22], we performed an in-depth analysis of rodent ACRC orthologs and showed that many rodent species possess a SprT-like domain; it will be interesting to determine why it has been lost during evolution, specifically in certain mouse and rat species and how this impacted ACRC function in those species (Fig. 1A, Supplementary Fig. S1–S3, and Supplementary Table S1). We have also shown that the 3D structural models of the SprT-like domain of the human and zebrafish ACRC orthologs are nearly identical (Fig. 1B), making zebrafish a good model for studying ACRC-mediated DPC proteolysis.

ACRC was originally described as a protein with a specific role in germ cells [23] [22, 24, 25]. In line with that, we found very high and dominant expression of *acrc* in both ovaries and testes (Fig. 2A). Interestingly, we found that *acrc* is also expressed in all other tissues analyzed (Fig. 2A). In contrast, we confirmed that mouse *Acrc* is expressed almost exclusively in the testis (see Gene Expression Database for Gcna under www.informatics.jax.org, February, 2023, and Fig. 2D), which further highlights the difference between mouse Acrc which lacks a SprT-like domain and other vertebrate orthologs, zebrafish included (Fig. 1B). Of note, the *ACRC* gene expression pattern in humans is more similar to zebrafish *acrc* expression pattern: unlike in mouse, human and zebrafish *ACRC* are expressed in adult somatic tissues [65]. Considering the substantial mRNA expression levels of *ACRC* in adult tissues of zebrafish and humans, we suggest it plays a role beyond the DPC repair in meiosis in the germline, as was previously postulated [22, 24, 25].

Comparison of the expression levels of *acrc* and *sprtn* during zebrafish embryonic development showed that both genes are highly expressed up to 6 hpf, but also that *acrc* is expressed at least three-fold more strongly than *sprtn* between 1 and 48 hpf, suggesting that Acrc is the dominant DPCR protease at these stages (Fig. 2C). This is supported by our results showing that Acrc is absolutely required during embryonic development (Fig. 3).

**Figure 7.**
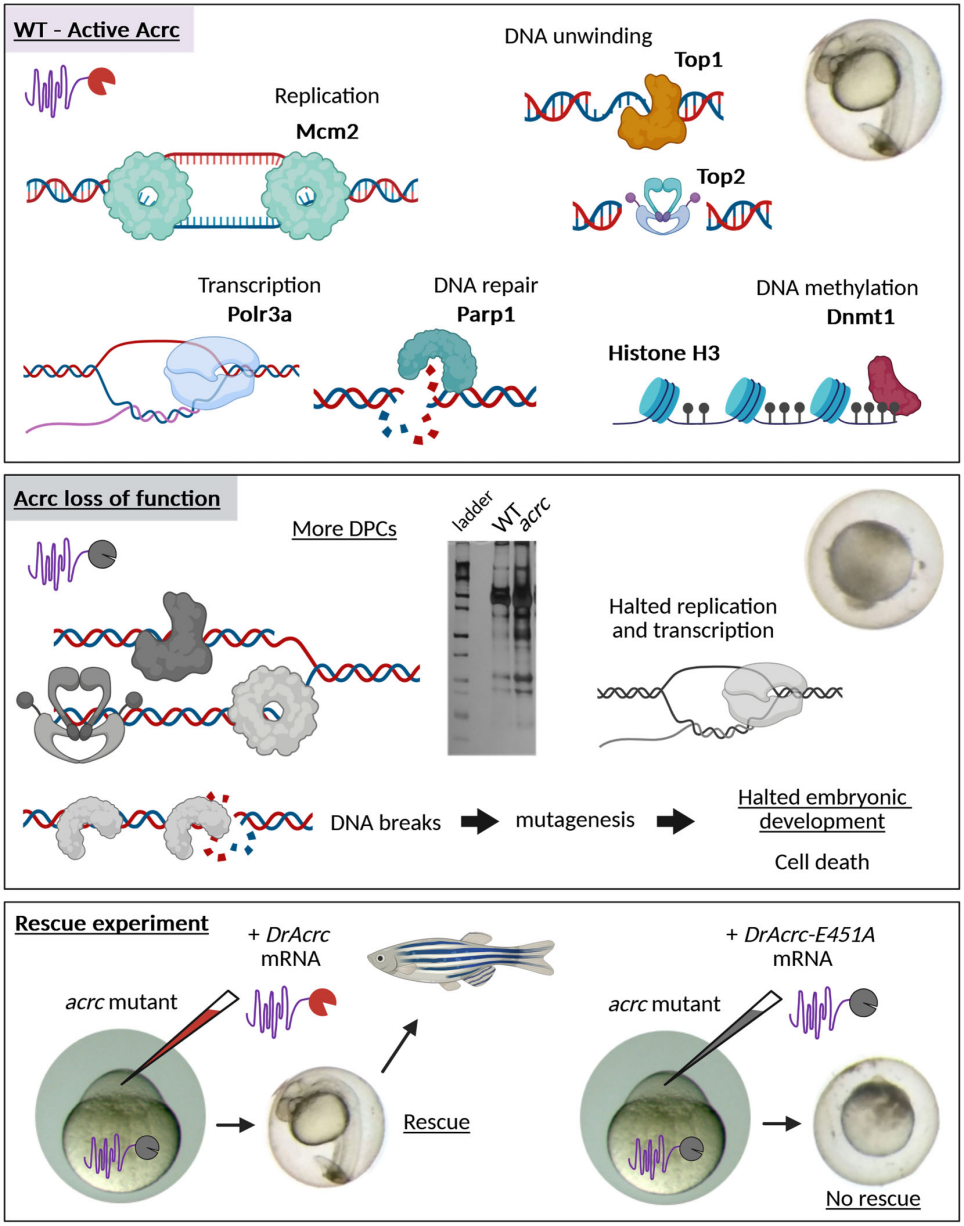
Model of Acrc-mediated DPC repair during embryonic development. In WT embryos, Acrc repairs multiple cellular DPCs during rapid cell divisions, DNA methylation and intense transcription. When the protease core of Acrc is mutated (Acrc loss of function), DPCs accumulate and embryos die before 24 hpf, probably due to arrested cell division. In contrast, when Acrc is supplemented at the one-cell stage by injection of mRNA encoding Acrc-WT into *acrc* mutant embryos, development proceeds and fish carrying a mutation in the acrc *gene* can develop to adulthood. On the contrary, when the catalytic mutant form of Acrc is supplemented, development cannot progress. Created in BioRender. Otten, C. (2026) https://BioRender.com/34fnyw6.

We showed that the intact protease core of Acrc is essential for embryonic survival, as the mutant line with a deletion of the catalytic glutamate (E451) and three downstream amino acids (*rbi5* allele, Fig. 3A and B) exhibited embryonic lethality (Fig. 3F and G). Previously, it was observed that the absence of the entire Acrc protein is lethal in zebrafish [25], but it was not known which domains of Acrc are responsible for this phenotype. Considering that *acrc* mutants can fully recover after injection of Acrc-WT mRNA into embryos at the one-cell stage and reach adulthood without obvious defects, we suggest that Acrc is the only DPCR factor that can repair specific DPC lesions at those early developmental stages (Fig. 7). Other DPC proteases or nucleolytic DPC repair pathways present at this developmental stage under physiological conditions, cannot prevent embryonic death caused by deficiency in the protease core of Acrc. In contrast, Acrc is not essential in adults, as rescued *acrc* mutants develop normally and adult fish do not show visible differences compared with WT fish.

Our flexible nongenetic rescue system allows us to rapidly test different constructs encoding mutant forms or deletions of Acrc in a vertebrate model organism. Here, we used this system to examine the function of individual domains of Acrc. First, we confirmed the results from the *rbi5* (ΔEMCH) mutant line showing that the catalytic function of Acrc is essential, because injection of *acrc* mRNA with the E451A mutation did not rescue the lethality phenotype (Fig. 4C and Supplementary Fig. S6C). Previously, a similar experiment was performed in *Drosophila* using transgenic expression of WT and HE > AA mutant Acrc/Gcna in the mutant background [25] which showed that dsDNA breaks were not rescued by the HE > AA mutant, while egg hatching impairment was rescued, thereby suggesting the function for Acrc/Gcna besides its catalytic activity in flies. Using this separation-of-function approach, we found that deletion of the SprT-like domain impairs the function of Acrc and that the IDR domain alone cannot rescue the lethality phenotype, whereas injection of the SprT-like domain alone can (Fig. 4C, and Supplementary Figs S6 and S7). This suggests that the Sprt-domain of Acrc and not the IDR domain is essential for development. We additionally confirmed these findings by injecting mouse *Acrc* mRNA which consists only of an IDR region (Fig. 4D, and Supplementary Figs S6B and C, and S10A). In comparison, an *Acrc* conditional KO mouse model showed defects in spermatogenesis including dsDNA breaks and defective chromatin compaction, possibly via the TOP2 interaction, but a direct role of mouse ACRC in DPCR has not been investigated. Instead, it has been proposed that mouse ACRC promotes genome integrity during meiosis by mediating protein-protein interactions via its IDR domain and its SUMO-interaction motifs [24] and by acting as a histone chaperone [26]. We showed that Sprtn and Acrc are not redundant, considering that *sprtn* cannot overcome Acrc deficiency in the early development, despite the fact that *sprtn* is endogenously highly expressed at 6 hpf in *acrc* mutants (Fig. 2C, and Supplementary Fig. S10G and H) and the fact that injection of additional *sprtn* mRNA could not compensate for the lethality phenotype (Fig. 4D, and Supplementary Figs S6B and C, and S10A). Our results offer a direct functional proof that Sprtn cannot compensate for the deficiency in the protease core of Acrc during development. The reason for this probably lies in different expression patterns throughout the cell cycle, as it was previously shown that Sprtn is degraded during mitosis and is present in S and G2 [66], while Acrc levels are lowest in G1, increase through S, and remain high in G2, and Acrc is especially enriched on condensed mitotic chromosomes [24]. Because a catalytic mutation in the protease core causes early embryonic lethality, we investigated whether DPC accumulation precedes the lethal phenotype. Indeed, endogenous DPC levels were significantly increased in mutant embryos at 6 hpf, when they still exhibited a normal WT-like phenotype (Figs 5 and 6, and Supplementary Fig. S11), suggesting a link between DPC accumulation and lethality. Specifically, Top1-, Top2-, and Parp1-DPCs accumulated to a high degree, followed by substantial accumulation of histone H3-DPCs, and moderate accumulation of Dnmt1-, Polr3a-, and Mcm2-DPCs (Fig. 6A and B, and Supplementary Fig. S11C and D). All the latter proteins are involved in essential cellular processes: TOP1, TOP2, and MCM2 in DNA replication, PARP1 in DNA repair and transcription, RNA Pol III, TOP1, and TOP2 in transcription, and DNMT1 in maintaining DNA methylation patterns following DNA replication (Fig. 7). Considering the very high accumulation of these and other DPCs as a consequence of Acrc deficiency, and the fact that other factors including Sprtn, the proteasome and nucleases cannot compensate for the lack of Acrc in the embryos, we conclude that ACRC is an essential putative DPC protease in the early development (Fig. 7). We propose that ACRC is the main factor removing DPCs in mitosis during the development, as we show that SPRTN cannot rescue viability of zebrafish embryos. Cellular processes change dramatically during embryonic development. In zebrafish, the fertilized egg initially develops using maternally deposited mRNAs and proteins until the maternal-zygotic transition occurs around 3–4 hpf, leading to zygotic genome activation [67]. Additionally, during this time, cells divide every 30 min and are always in S-phase, which means that many DNA transactions occur simultaneously, such as DNA unwinding, transcription, replication, and heterochromatin formation, which in turn makes DNA highly susceptible to DPC formation. This is a critical stage in embryonic development, when unperturbed mitosis is essential for survival, so the fidelity of DNA repair is crucial, and we suggest that ACRC plays an essential role in this process. While this paper was under revision, a study by Tomaskovic *et al.* (2026) was published, showing that SPRTN can remove DPCs during mitosis in human cells in culture and in a primary liver cancer cell line from the *Sprtn*  ^Y118C/Y118C^ mouse [68]. In light of these new findings, we propose that ACRC with SprT domain is the dominant mitotic DPC protease, particularly essential during rapid embryonic cell divisions when the mitosis-to-interphase ratio is maximized, while SPRTN contributes to mitotic DPC repair in cells with longer cell cycles. We hypothesize that SPRTN cannot compensate for the loss of functional ACRC during early zebrafish development because it is repeatedly degraded by APC-Cdh1 at mitotic exit, with insufficient time for re-accumulation during rapid divisions, and because the DPC load is too high in mitotic embryonic cells without functional ACRC for SPRTN alone to manage. In mouse, where ACRC has lost the SprT domain, SPRTN has likely assumed a more prominent mitotic role in DPC repair by necessity [68].

## Supplementary Material

gkag324_Supplemental_File

## Data Availability

Protein multiple sequence alignments used to build the phylogenetic trees are available at Figshare under DOI: 10.6084/m9.figshare.30884879; details of rescue experiments are show in a file available at Figshare under DOI: 10.6084/m9.figshare.30920183.

## Notes

Present address: Lee Kong Chian School of Medicine (LKC Medicine), Nanyang Technological University, Singapore636921, Singapore
