# Supplementary material for: ACRC/GCNA is an essential protease that repairs DNA–protein crosslinks during vertebrate development
Source: Nucleic Acids Res. 2026 Apr 20;54(7):gkag324. doi: 10.1093/nar/gkag324 (PMC13092972; doi:10.1093/nar/gkag324)
Supplement: gkag324_Supplemental_File [file gkag324_supplemental_file.pdf]

ACRC/GCNA is an essential protease that repairs DNA-protein crosslinks during vertebrate development

Cecile Otten, Marin Kutnjak, Christine Supina-Pavic, Marija Pranjic, Ivan Anticevic, Vanna Medved, Marta Popovic

- **Supplemental Figures S1 - S11**
- **Supplemental Tables S1 - S3**

Figure S1

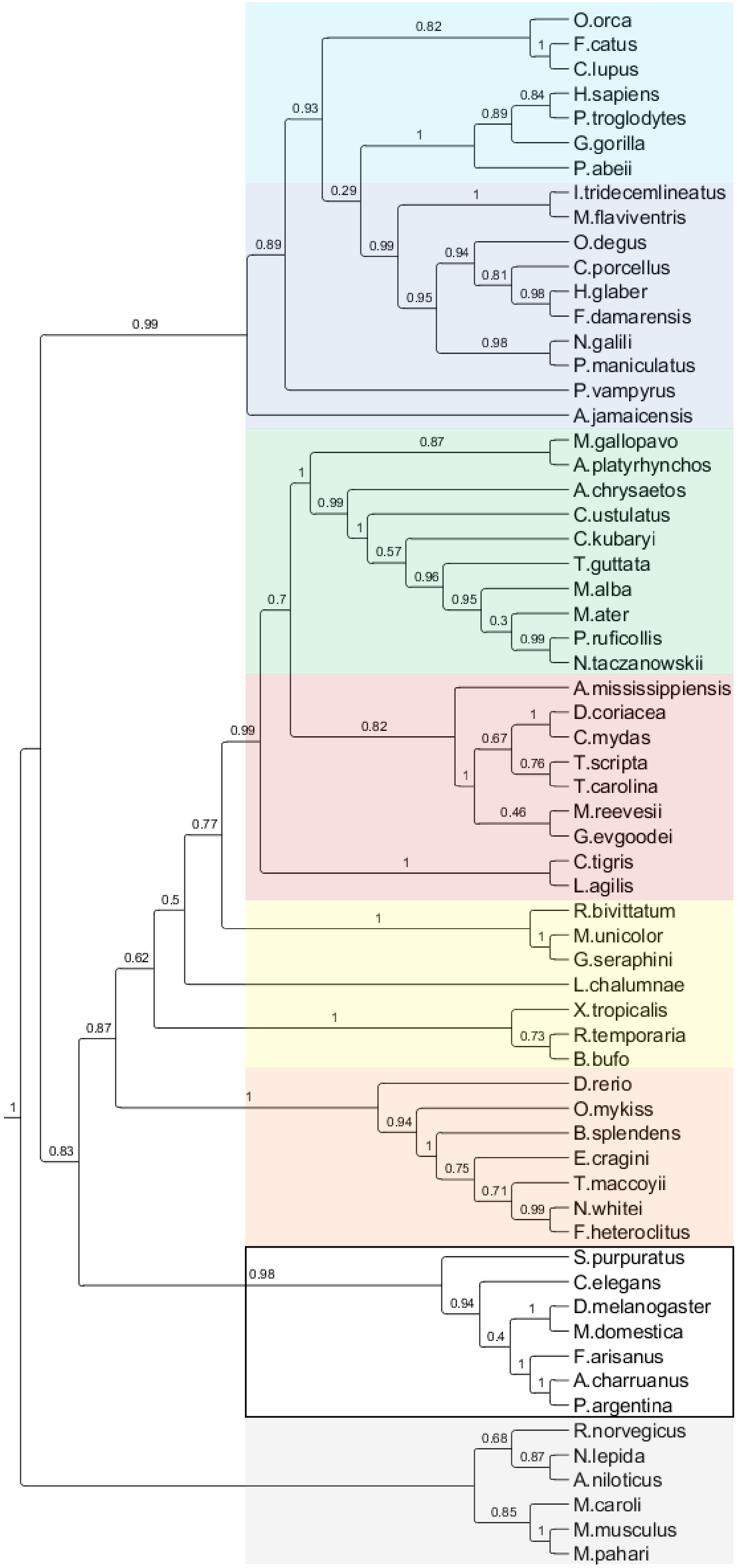

Figure S1. Extended phylogenetic tree of ACRC/GCNA orthologs based on the full-length protein sequences. Related to Figure 1A.

Figure S2

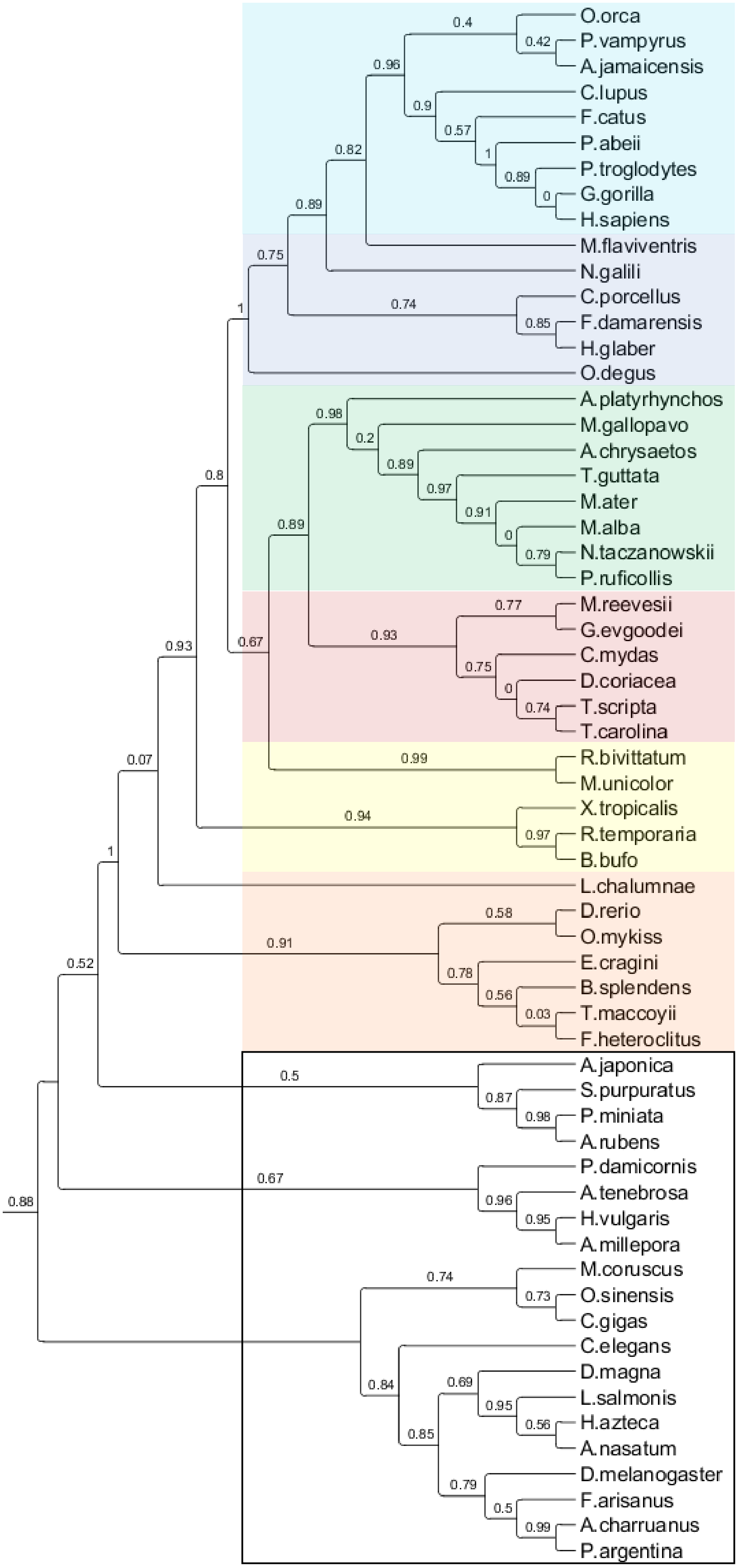

Figure S2. Extended phylogenetic tree of ACRC/GCNA orthologs based on the SprT-like domains. Related to Figure 1A.

Figure S3

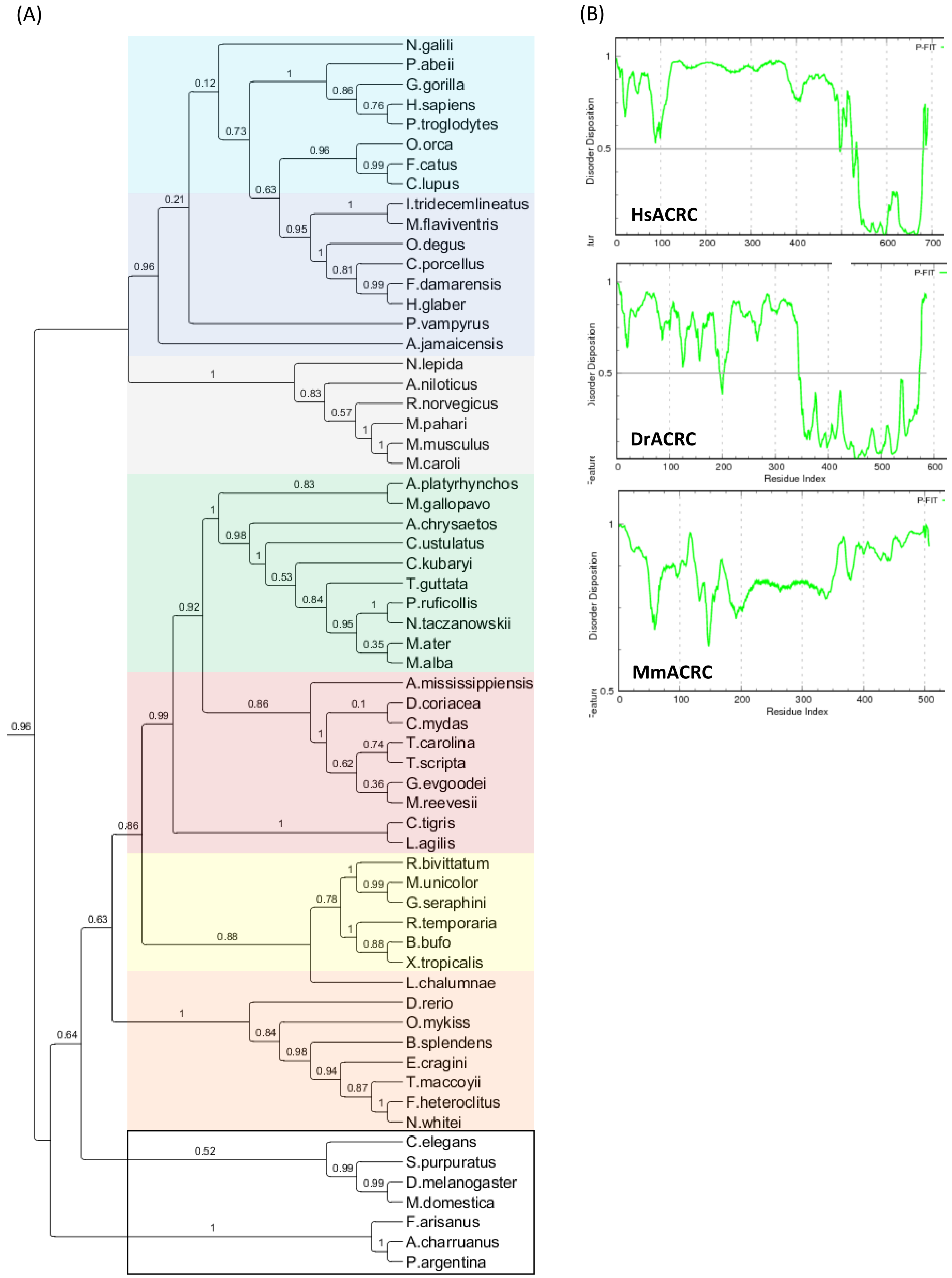

Figure S3. (A) Extended phylogenetic tree of ACRC/GCNA orthologs based on the N-terminal / non-structured domains. **(B)** Disorder disposition plot for human, zebrafish and mouse ACRC predicted using PONDR-FIT workspace. Related to Figure 1A.

Figure S4

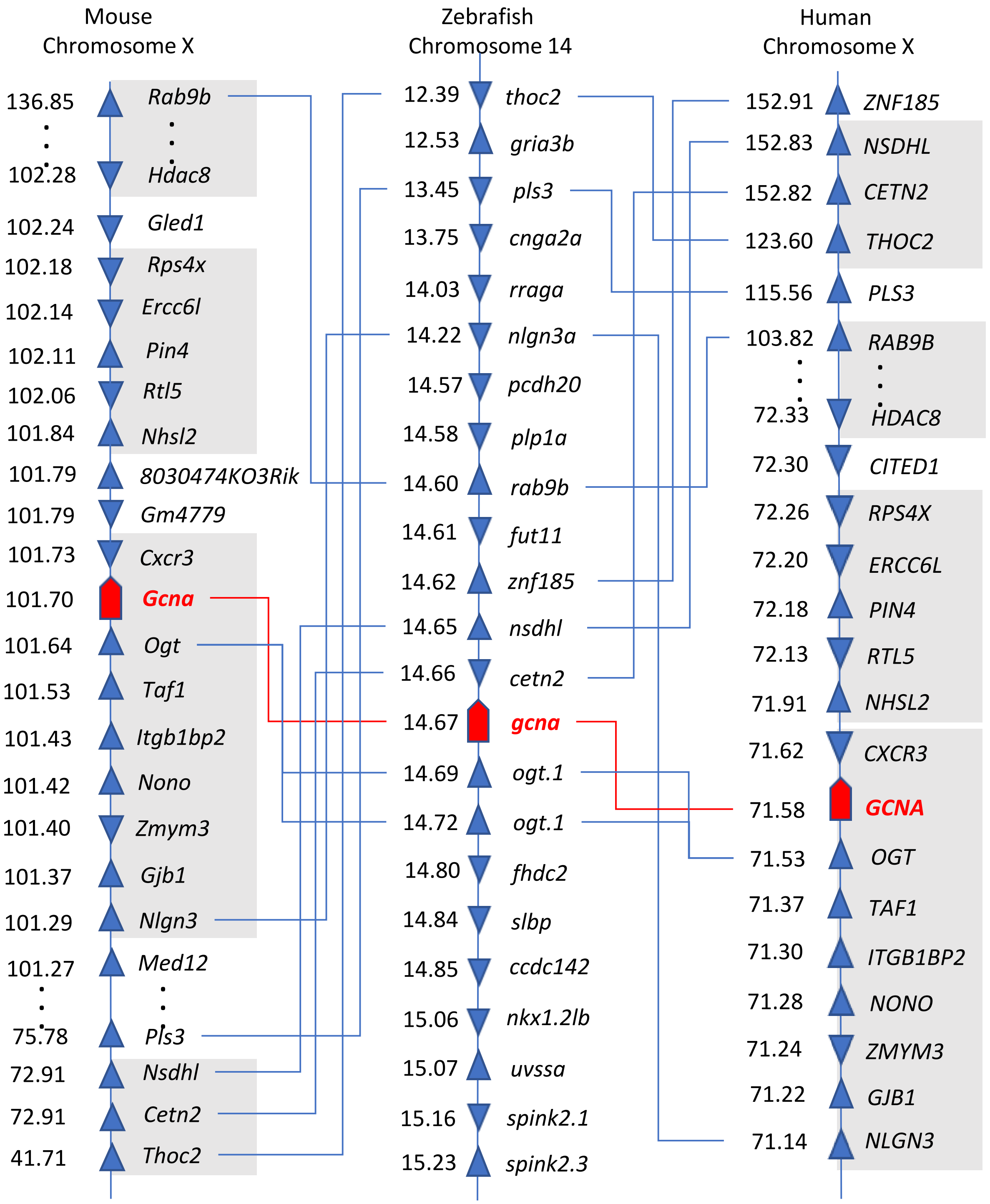

Figure S4. Conserved synteny analysis of zebrafish, mouse and human *ACRC/GCNA* genes.

Figure S5

(A)

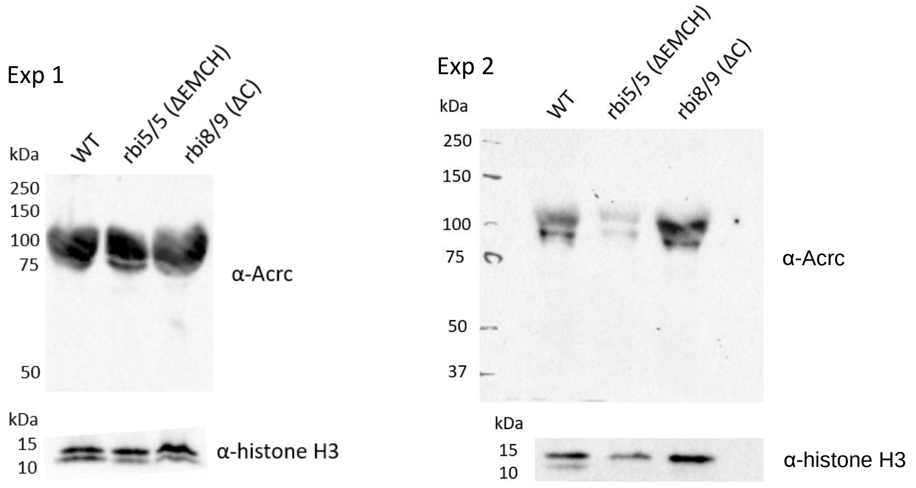

(B)

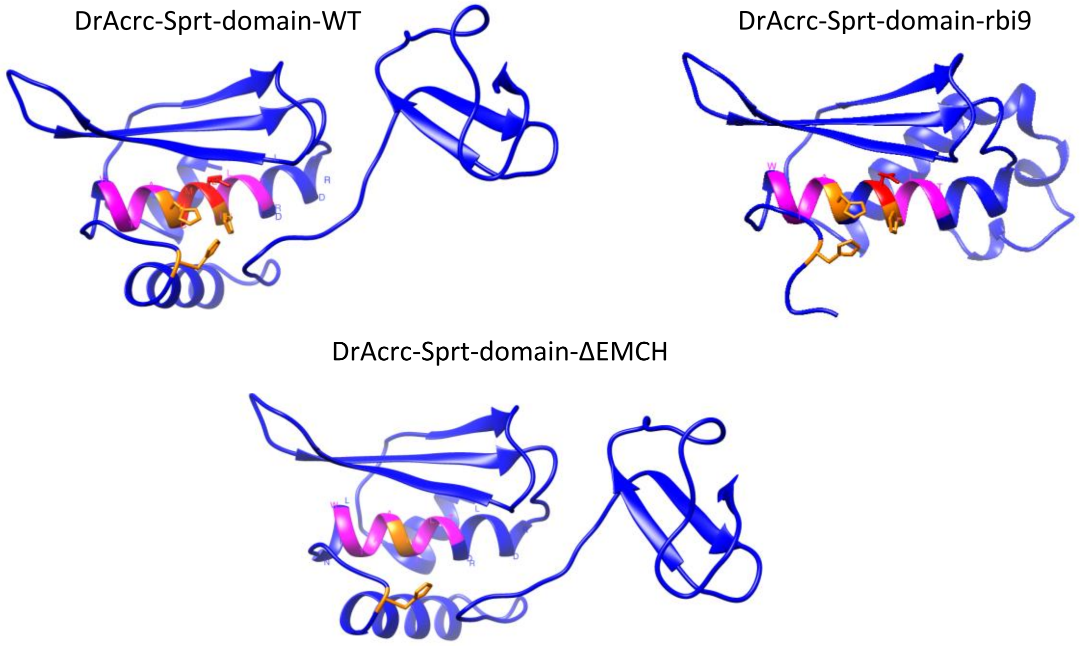

(C)

	WT	ΔEMCH	Difference to WT	ΔEMCH	rbi9	Difference to WT	rbi9
	kcal/mol	kcal/mol	kcal/mol	Predicitive effect	kcal/mol	kcal/mol	Predicitive effect
Backbone hydrogen bonds	-86,77	-76,92	+9,85	destabilizing	-53,30	+33,47	destabilizing
Sidechain hydrogen bonds	-17,28	-15,32	+1,96	destabilizing	-7,69	+9,59	destabilizing
Van der Waals energy	-164,75	-158,51	+6,24	destabilizing	-85,34	+79,41	destabilizing
Electrostatic energy	-4,22	-5,55	-1,33	stabilizing	-1,98	+2,24	destabilizing
Polar solvation energy	226,34	223,08	-3,26	stabilizing	120,43	-105,91	stabilizing
Hydrophobic solvation energy	-209,60	-201,16	+8,44	destabilizing	-107,34	+102,26	destabilizing
Van der Waals clash	39,89	48,06	+8,17	destabilizing	30,75	-9,14	stabilizing
Torsional strain energy	3,94	4,05	+0,11	destabilizing	3,70	-0,24	stabilizing
Backbone van der Waals clash	94,17	90,24	-3,93	stabilizing	61,24	-32,93	stabilizing
Sidechain conformational entropy	81,19	75,67	-5,52	stabilizing	42,56	-38,63	stabilizing
Mainchain conformational entropy	225,47	219,06	-6,41	stabilizing	134,67	-90,8	stabilizing
Water bonds energy	0,00	0,00	0	no effect	0,00	0	no effect
Helix dipole energy	-1,70	-2,02	-0,32	stabilizing	-0,41	+1,29	destabilizing
Loop conformational entropy	0,00	0,00	0	no effect	0,00	0	no effect
Cis-peptide bond energy	0,00	0,00	0	no effect	0,00	0	no effect
Disulfide bond energy	0,00	0,00	0	no effect	0,00	0	no effect
Knowledge-based electrostatic interactions	0,00	0,00	0	no effect	0,00	0	no effect
Partial covalent interaction energy	0,00	0,00	0	no effect	0,00	0	no effect
Ionization energy of charged residues	1,12	0,94	-0,18	stabilizing	0,46	-0,66	stabilizing
Complex entropy penalty	0,00	0,00	0	no effect	0,00	0	no effect
Total	93,63	111,4	+17,77	destabilizing	76,51	-17,12	stabilizing

Figure S5. (A) Western blot analysis of Acrc protein levels in mutant and WT fish shows the presence of zebrafish Acrc protein in WT fish, and both mutant strains: *acrc*$^{rbi5/rbi5}$ (ΔEMCH) and *acrc*$^{rbi8/rbi9}$ in lysates of adult ovaries. The size of the WT protein is 65.43 kDa, the ΔEMCH form is 64.94 kDa and the truncated ACRC in *acrc*$^{rbi8/rbi9}$ mutants is 52.56 kDa. The Acrc protein is visible at an apparent molecular weight of 100 kDa due to its acidic/hydrophilic composition and IDR region, which causes a gel shift as explained in the results. **(B)** Structural model of zebrafish Acrc-Sprt domain-WT and **(C)** Acrc-Sprt domain- ΔEMCH (*acrc*$^{rbi5/rbi5}$ mutant line). Models were built using ColabFold (AlphaFold) and visualized with Chimera. The residue confidence score, pLDDT, was > 90 (very high, for each residue shown in the model). The deleted residues, EMC are shown in red; Zn-bearing histidines in orange; adjacent residues within the α-helix in magenta, and all other residues in blue. **(C)** Energy parameters (kcal/mol) for the Sprt domain of Acrc-WT, ACRC-ΔEMCH and ACRC with a premature stop at amino acid 472 (rbi9 allele) were calculated using FoldX. Differences between the mutants and WT form are shown (mutant minus WT), where a negative change indicates possible stabilization, and a positive change indicates possible destabilization.

Figure S6

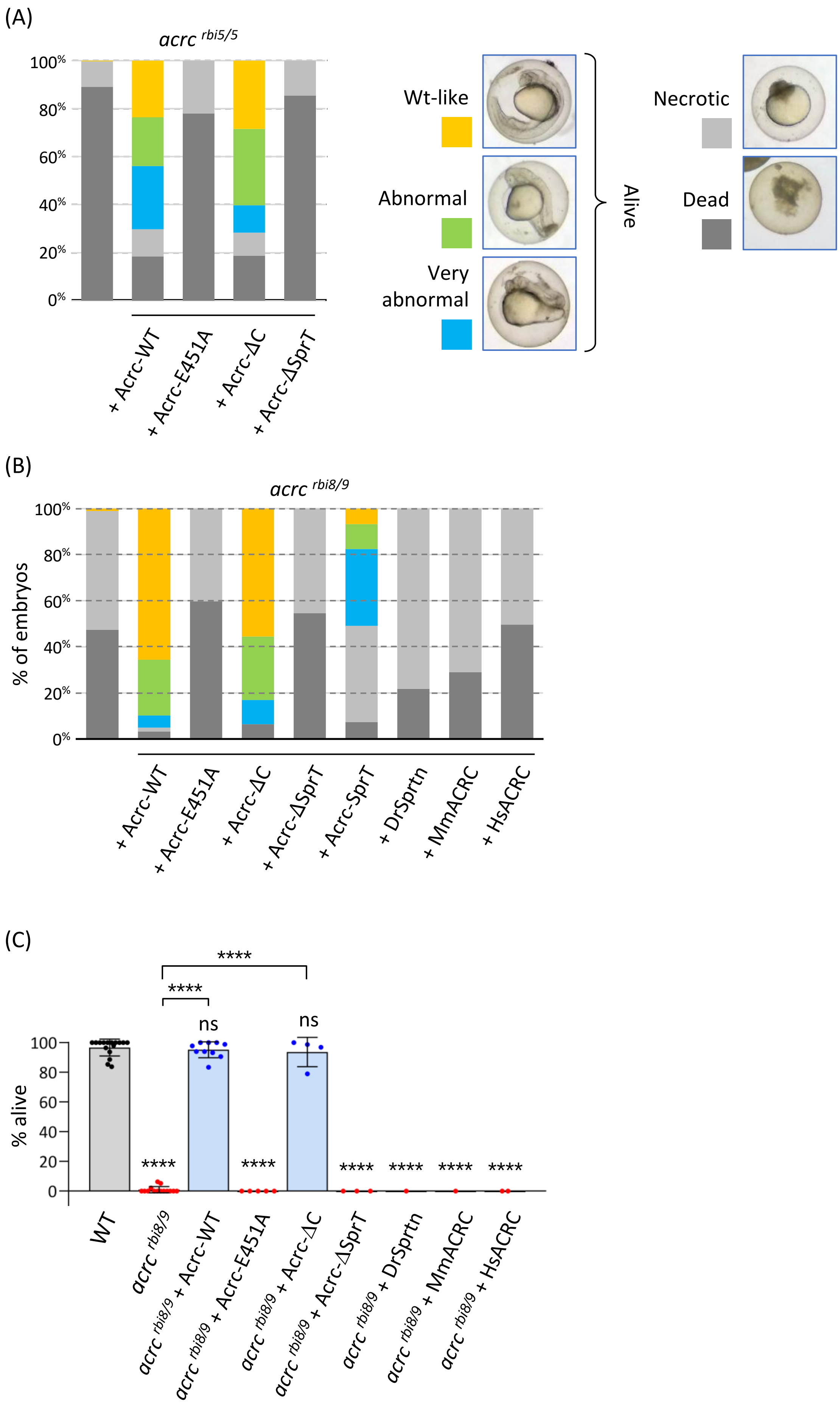

(A)
acrc rbi5/5
100%
80%
60%
40%
20%
0%
+ Acrc-WT
+ Acrc-E451A
+ Acrc-ΔC
+ Acrc-ΔSprT
Wt-like
Abnormal
Very abnormal
Alive
Necrotic
Dead
(B)
acrc rbi8/9
% of embryos
+ Acrc-WT
+ Acrc-E451A
+ Acrc-ΔC
+ Acrc-ΔSprT
+ Acrc-SprT
+ DrSprtn
+ MmACRC
+ HsACRC
(C)
% alive
****
ns
WT
acrc rbi8/9
acrc rbi8/9 + Acrc-WT
acrc rbi8/9 + Acrc-E451A
acrc rbi8/9 + Acrc-ΔC
acrc rbi8/9 + Acrc-ΔSprT
acrc rbi8/9 + DrSprtn
acrc rbi8/9 + MmACRC
acrc rbi8/9 + HsACRC

Figure S6. Detailed phenotype quantification of *acrc*$^{rbi5/rbi5}$ and of *acrc*$^{rbi8/rbi9}$ embryos after injection of various zebrafish Acrc mRNA constructs, Sprtn mRNA, and mouse and human Acrc mRNAs, related to main Figure 4. **(A-B)** Graphical representation of the phenotype range observed after injection of *acrc*$^{rbi5/rbi5}$ (A) or *acrc*$^{rbi8/rbi9}$ (B) embryos with the mRNA constructs shown in Figure 4A at 24 hpf. Each embryo was categorized according to the severity of the phenotype (see Materials and Methods). **(C)** Quantification of survival of *acrc*$^{rbi8/rbi9}$ embryos at 24 hpf derived from the data shown in (B). Data are presented as the percentage of live embryos in a sample (n > 14) from independent experiments (n = 2-9) (mean ± SD). Statistically significant differences compared to WT are indicated by asterisks above each bar in the graph (one-way ANOVA and Dunnett's tests, **** p-value < 0,0001; *** p-value = 0,0005; ns: not significant).

Figure S7

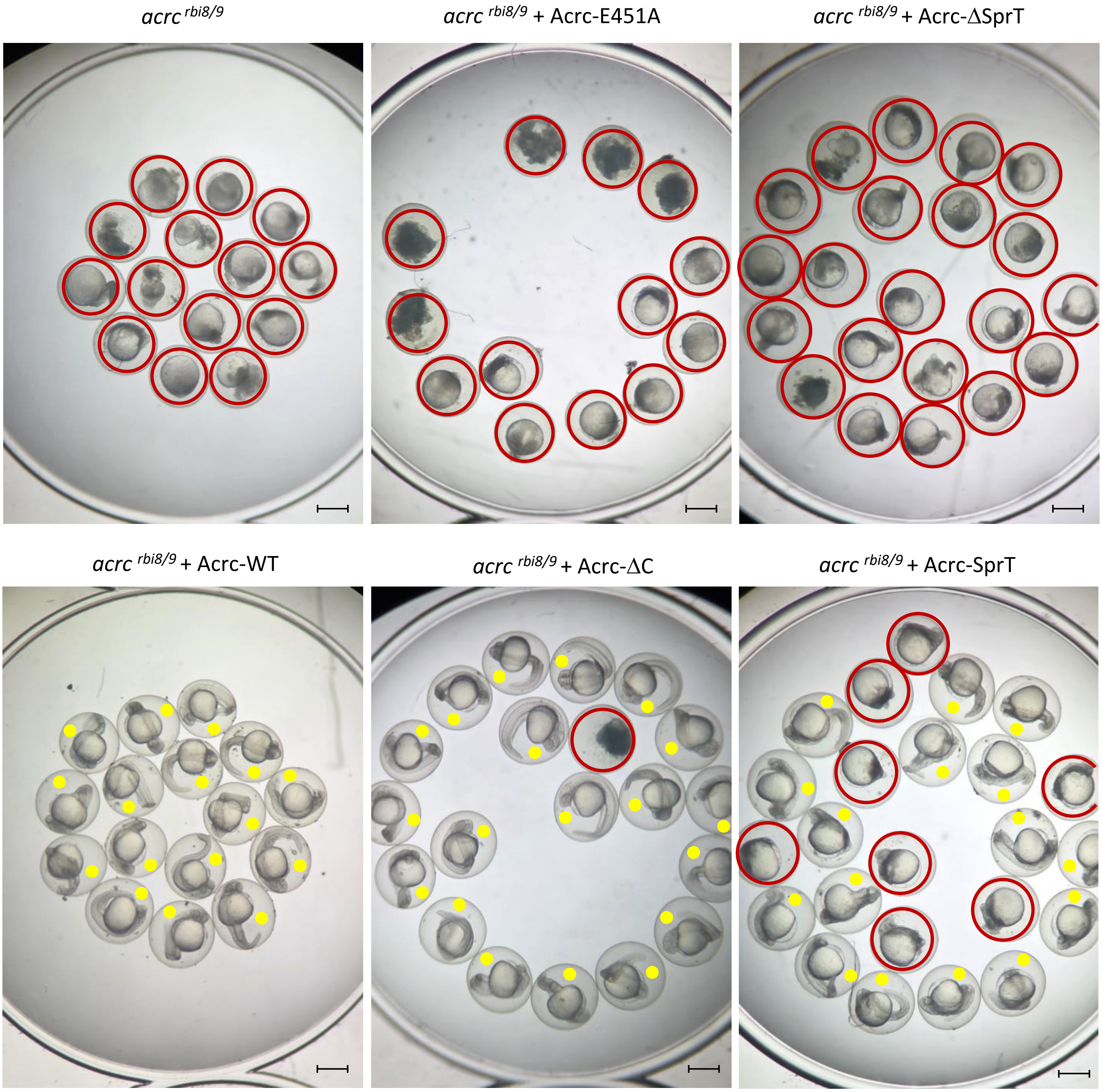

Figure S7. Representative images of *acrc*$^{rbi8/rbi9}$ mutant embryos at 24 hpf, injected with the following rescue constructs that do not rescue the phenotype: Acrc-E451A and Acrc-DSprT, and with constructs that do rescue the phenotype: Acrc-WT, Acrc-DC and Acrc-SprT. Scale bars: 500µm. Red circles indicate dead embryos; yellow dots indicate live embryos.

Figure S8

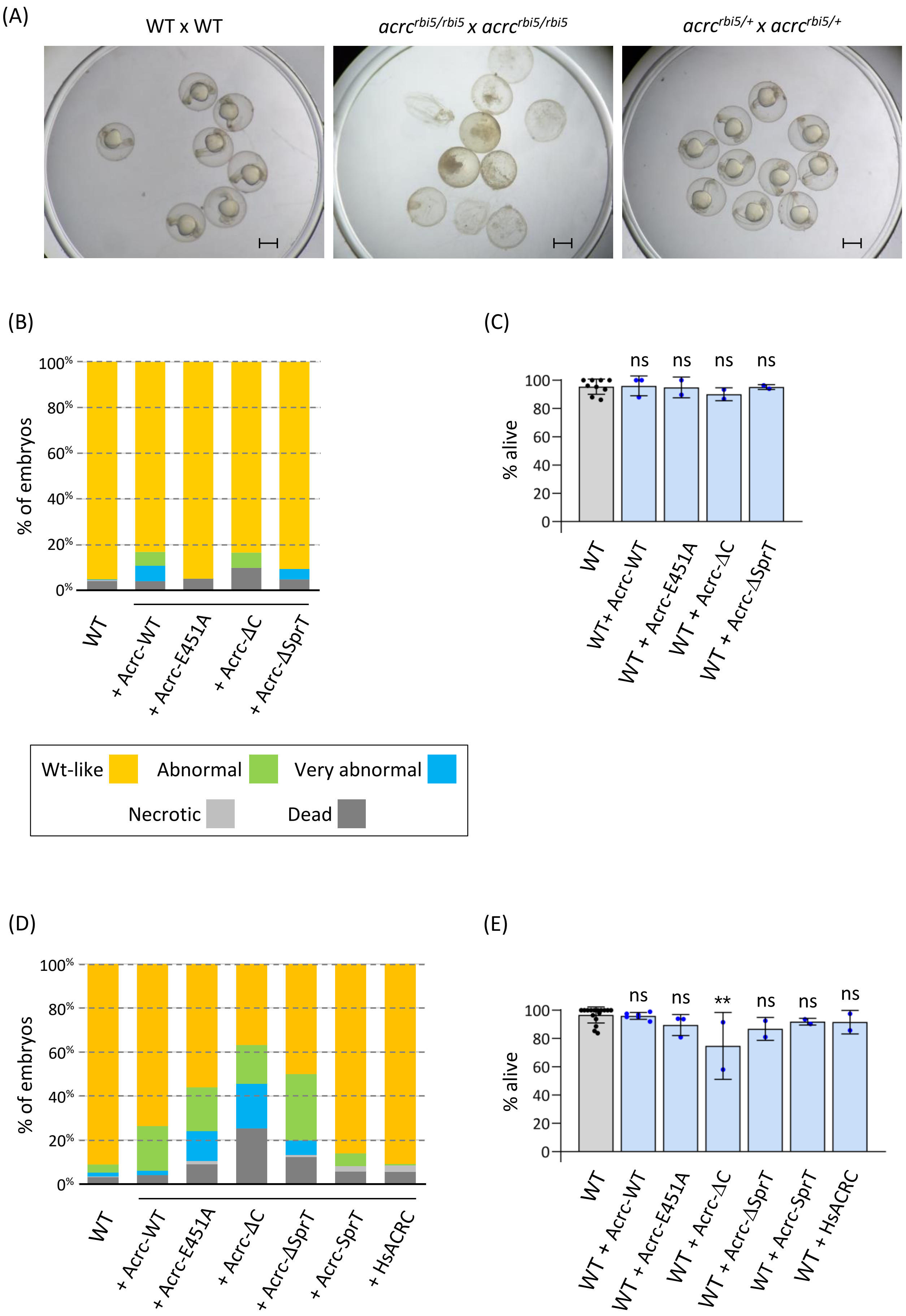

(A)
WT x WT
acrc rbi5/rbi5 x acrc rbi5/rbi5
acrc rbi5/+ x acrc rbi5/+
(B)
100%
80%
60%
40%
20%
0%
% of embryos
WT
+ Acrc-WT
+ Acrc-E451A
+ Acrc-ΔC
+ Acrc-ΔSprT
(C)
ns
ns
ns
ns
100
80
60
40
20
0
% alive
WT
WT+ Acrc-WT
WT + Acrc-E451A
WT + Acrc-ΔC
WT + Acrc-ΔSprT
Wt-like
Abnormal
Very abnormal
Necrotic
Dead
(D)
100%
80%
60%
40%
20%
0%
% of embryos
WT
+ Acrc-WT
+ Acrc-E451A
+ Acrc-ΔC
+ Acrc-ΔSprT
+ Acrc-SprT
+ HsACRC
(E)
ns
ns
**
ns
ns
ns
100
80
60
40
20
0
% alive
WT
WT + Acrc-WT
WT + Acrc-E451A
WT + Acrc-ΔC
WT + Acrc-ΔSprT
WT + Acrc-SprT
WT + HsACRC

Figure S8. Detailed phenotype quantification of WT embryos, injected with rescue constructs. **(A)** Phenotype of zebrafish *acrc*$^{rbi5/rbi5}$ maternal zygotic mutants at 24 hpf obtained by crossing homozygous parents (middle panel, all dead), and mutants obtained by crossing heterozygous parents (right panel, all alive, indistinguishable from WT and heterozygous siblings). **(B)** Graphical representation of the phenotype range observed after injection of WT embryos with the following mRNA constructs: Acrc-WT, Acrc-E451A, Acrc-ΔC and Acrc-ΔSprt. **(C)** Quantification of WT embryo survival at 24 hpf derived from the data shown in (B). **(D)** Graphical representation of the phenotype range observed after injection of WT embryos with the following zebrafish mRNA constructs: Acrc-WT, Acrc-E451A, Acrc-ΔC, Acrc-ΔSprT, Acrc-SprT and human ACRC mRNA. **(E)** Quantification of WT embryo survival at 24 hpf derived from the data shown in (D). Shown are values (mean and SD) for individual experiments (n = 2-9, with at least 15 embryos per experiment). Statistically significant differences compared to WT were analysed with one-way ANOVA followed by Dunnett's test (ns, not significant; **p-value < 0.005).

Figure S9

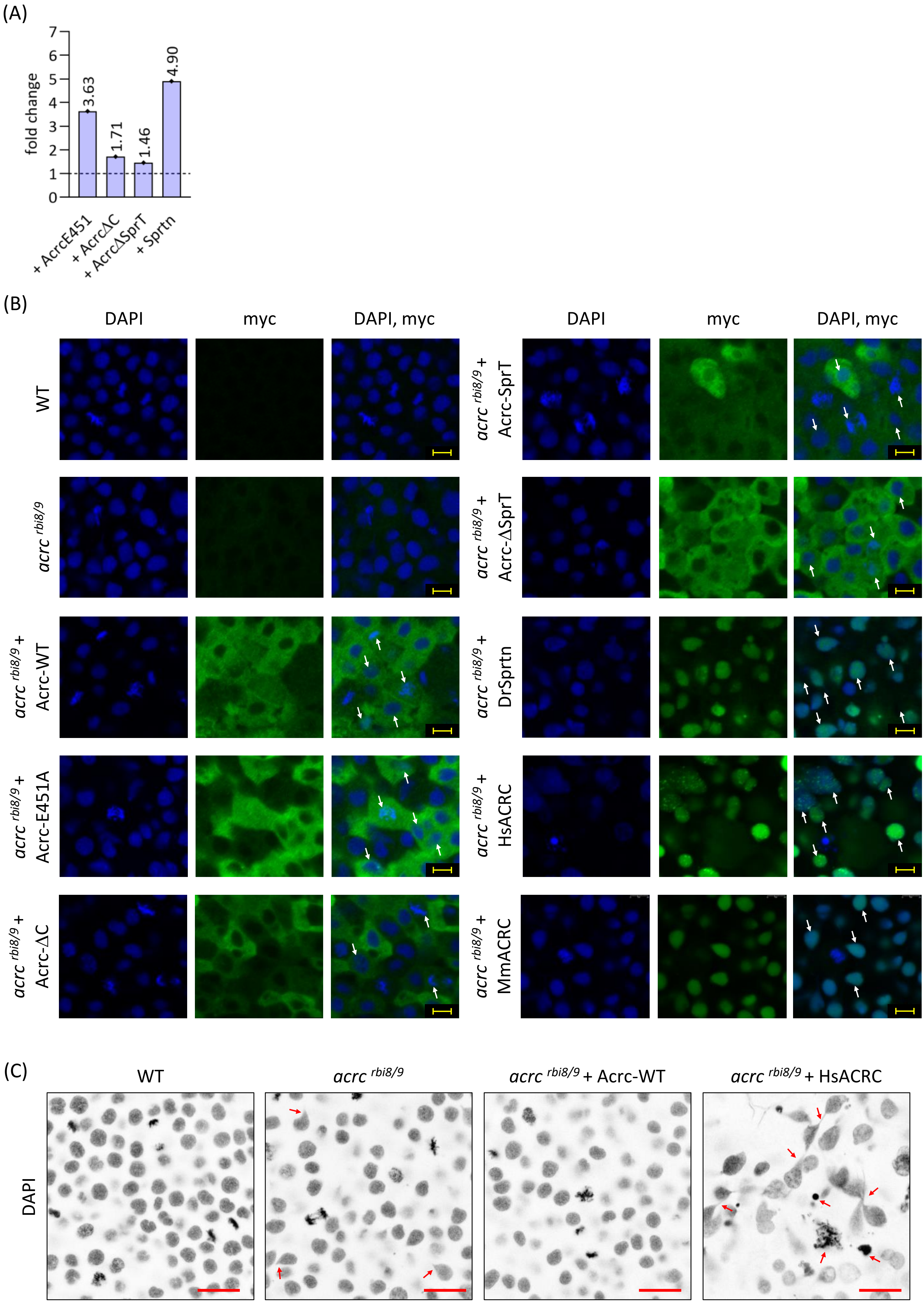
(A)
fold change
3.63
1.71
1.46
4.90
+ AcrcE451
+ AcrcΔC
+ AcrcΔSprT
+ Sprtn
(B)
DAPI
myc
DAPI, myc
WT
acrc rbi8/9
acrc rbi8/9 + Acrc-WT
acrc rbi8/9 + Acrc-E451A
acrc rbi8/9 + Acrc-ΔC
acrc rbi8/9 + Acrc-SprT
acrc rbi8/9 + Acrc-ΔSprT
acrc rbi8/9 + DrSprtn
acrc rbi8/9 + HsACRC
acrc rbi8/9 + MmACRC
(C)
WT
acrc rbi8/9
acrc rbi8/9 + Acrc-WT
acrc rbi8/9 + HsACRC
DAPI

Figure S9. Verification of the expression of the rescue constructs. **(A)** Relative expression levels of injected mRNA shown as fold change compared to endogenous WT levels of each mRNA, determined by qPCR. **(B)** Representative confocal images of WT and *acrc*$^{rbi8/rbi9}$ mutant embryos at 6 hpf, either uninjected, or injected with each of the mRNA rescue construct used in this study; DAPI (blue) stains the nuclei, while myc (green) is a tag attached to each rescue construct. Note that all zebrafish Acrc-derived constructs have a similar subcellular localization, while DrSprtn, MmACRC and HsACRC colocalize exclusively in the nuclei. Yellow scale bars: 10 µm. White arrows indicate examples of DAPI and myc colocalization. **(C)** Representative confocal images of WT and *acrc*$^{rbi8/rbi9}$ mutant embryos at 6 hpf: uninjected or injected with zebrafish Acrc-WT or human ACRC (HsACRC); Dapi staining is shown as a black signal on a white background to highlight the abnormal nuclear shapes (red arrows); Red scale bars: 25 µm.

Figure S10

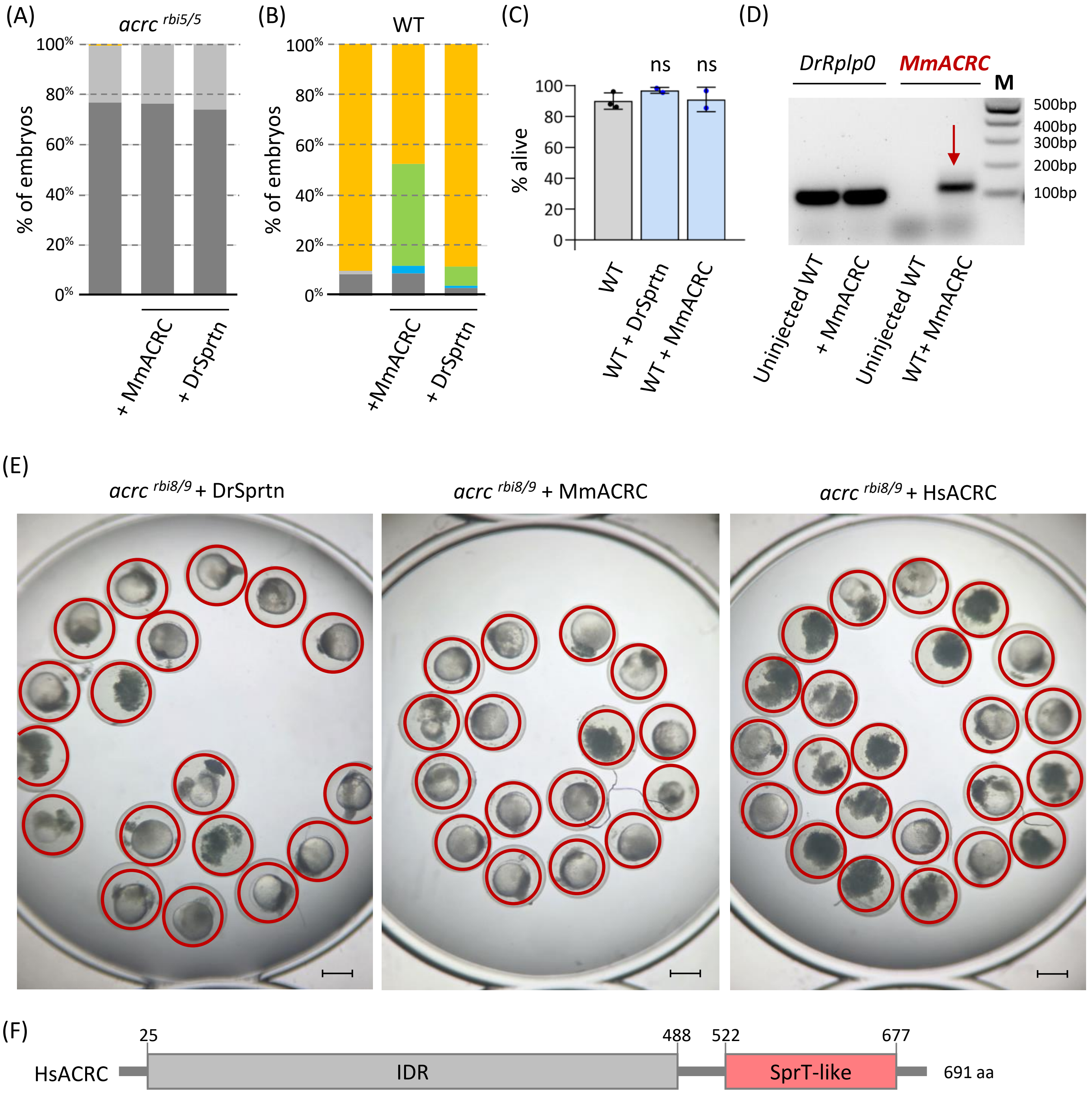

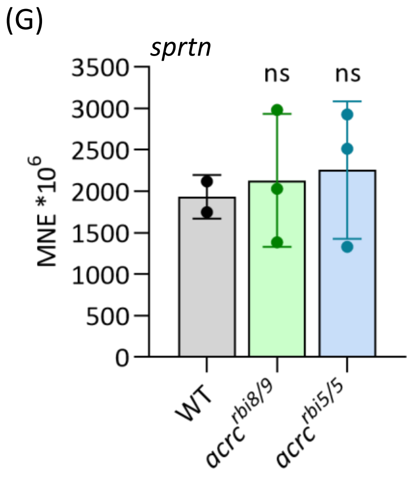

(H)

sprtn (6 hpf embryos)					
Genotype	Set1	Set2	Set3	Mean	SD
WT	1748	2118		1.933,0	261,6
$acrc^{rbi8/9}$	2979	1385	2031	2.131,7	801,8
$acrc^{rbi5/5}$	1329	2514	2926	2.256,3	829,1

Figure S10. Detailed phenotype quantification of *acrc*$^{rbi5/rbi5}$ embryos, injected with mouse Acrc mRNA (MmAcrc) and zebrafish Sprtn mRNA (DrSprtn). **(A-B)** Graphical representation of the phenotype range at 24 hpf, after injection into (A) *acrc*$^{rbi5/rbi5}$ and (B) WT embryos. **(C)** Quantification of WT embryo survival at 24 hpf derived from the data shown in (B). Values (mean and SD) for individual experiments are shown (n = 2-9, with at least 15 embryos per experiment). Differences to uninjected WT were not significant (ns, not significant, $p > 0{,}05$, one-way ANOVA and Dunnett's test). **(D)** Mouse Acrc expression after injection, determined by PCR (zebrafish *rplp0* as a control, M – DNA marker, Mm – *Mus musculus*). The red arrow indicates amplified MmACRC from injected embryos. **(E)** Representative images of *acrc*$^{rbi8/rbi9}$ mutant embryos at 24 hpf, uninjected and injected with DrSprtn, MmAcrc, or HsACRC mRNA. Scale bars: 500 µm. Red circles indicate dead embryos; yellow dots indicate live embryos. **(F)** Scheme of the HsACRC rescue construct injected into *acrc*$^{rbi8/rbi9}$ mutant embryos; Hs: *Homo sapiens*. **(G-H)** The relative expression level of *sprtn* is comparable between 6 hpf WT, *acrc*$^{rbi8/rbi9}$ and *acrc*$^{rbi5/rbi5}$ embryos, as quantified by qPCR. Data represent MNE (Mean Normalized Expression) ± SD normalized to the reference gene *elongation factor 1α* (*eef1a1l1*). Data (2 or 3 biological replicates of 10-25 pooled embryos each) were analyzed by one-way ANOVA followed by Dunnett's tests. Original data are shown in (H).

Figure S11

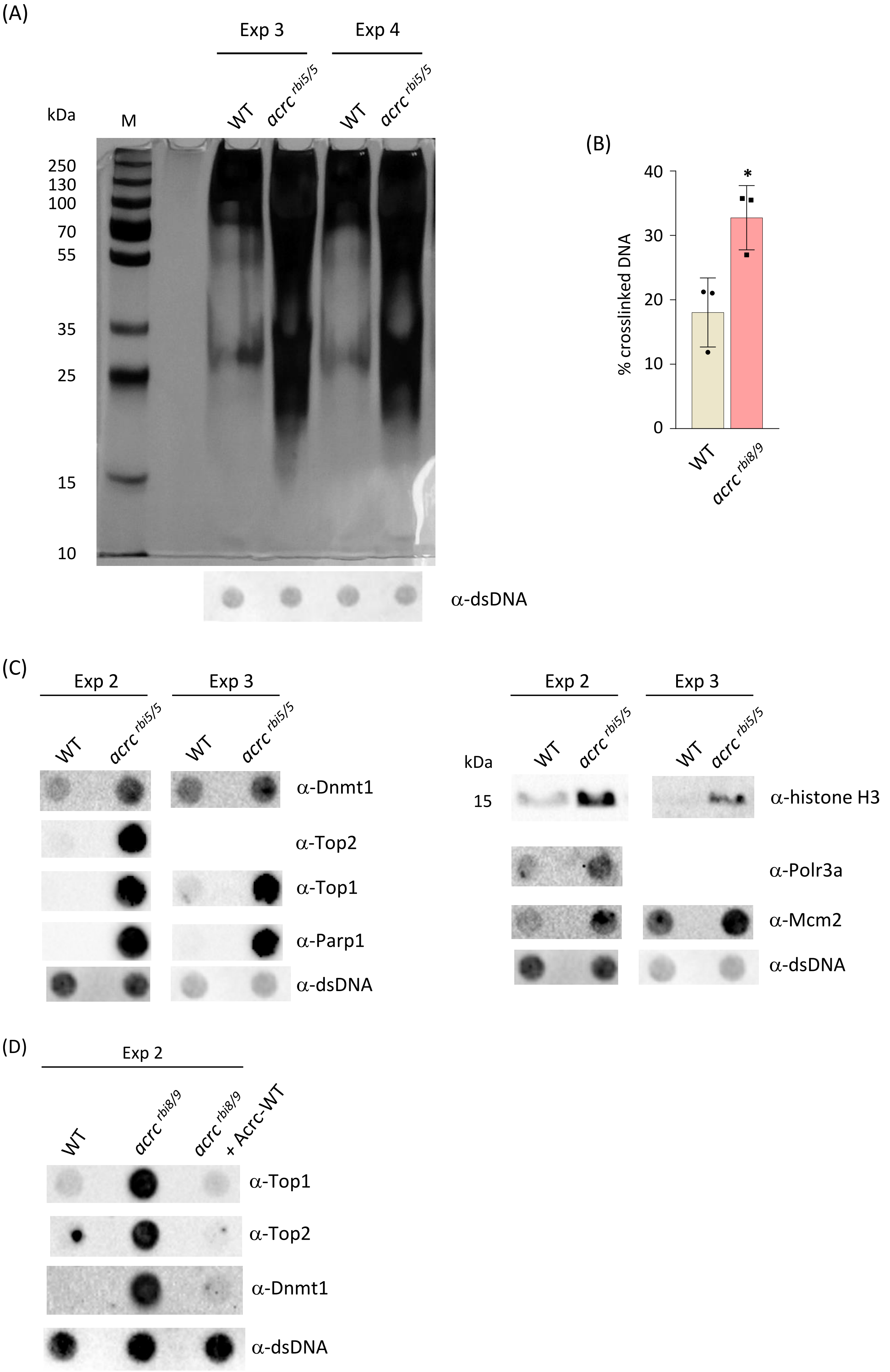
(A)
Exp 3
Exp 4
kDa
M
WT
acrc rbi5/5
250
130
100
70
55
35
25
15
10
α-dsDNA
(B)
% crosslinked DNA
40
30
20
10
0
*
WT
acrc rbi8/9
(C)
Exp 2
Exp 3
α-Dnmt1
α-Top2
α-Top1
α-Parp1
α-dsDNA
kDa
15
α-histone H3
α-Polr3a
α-Mcm2
(D)
Exp 2
acrc rbi8/9 + Acrc-WT
α-Top1
α-Top2
α-Dnmt1
α-dsDNA

Figure S11. (A) Total DPCs isolated using the modified RADAR method, from WT and *acrc*$^{rbi5/rbi5}$ mutant embryos (6 hpf), biological replicates 3 and 4 were used for quantification in Figure 5 (M – molecular weight marker). **(B)** Total DPCs (% of crosslinked DNA) isolated using the KCl/SDS method from *acrc*$^{rbi8/rbi9}$ mutant embryos are higher than in WT embryos at 6 hpf. An unpaired Student's t-test was performed for 3 biological replicates, each consisting of pools of 100 embryos each (*$p < 0.05$). **(C)** Specific DPCs analysed by dot-blot and detected by protein-specific primary antibodies in WT and *acrc*$^{rbi5/rbi5}$ embryos; biological replicates 2 and 3 (Exp 2 and Exp3) were used for quantifications in Figure 6B. **(D)** Specific DPCs analysed by dot-blot and detected by protein-specific primary antibodies in WT and *acrc*$^{rbi8/rb95}$ embryos; the second biological replicate (Exp 2), was used for the quantification in Figure 6D.

Table S1

Abbreviation	Accession no.	Species	Group
H.sapiens	NP_443189.1	Homo sapiens	mammals
G.gorilla	XP_018874375.2	Gorilla gorilla	mammals
P.troglodytes	NP_001258603.1	Pan troglodytes	mammals
P.abeii	XP_024096934.1	Pongo abelii	mammals
F.catus	XP_004000698.1	Felis catus	mammals
C.lupus	XP_038306204.1	Canis lupus	mammals
O.orca	XP_033294943.1	Orcinus orca	mammals
I.tridecemlineatus	XP_040137881.1	Ictidomys tridecemlineatus	mammals-rodents
M.flaviventris	XP_027791642.1	Marmota flaviventris	mammals-rodents
H.glaber	XP_021106213.1	Heterocephalus glaber	mammals-rodents
C.porcellus	XP_013009208.1	Cavia porcellus	mammals-rodents
P.maniculatus	XP_042124603.1	Peromyscus maniculatus bairdii	mammals-rodents
N.galili	XP_017650450.1	Nannospalax galili	mammals-rodents
F.damarensis	XP_033616375.1	Fukomys damarensis	mammals-rodents
O.degus	XP_023562888.1	Octodon degus	mammals-rodents
P.vampyrus	XP_023393196.1	Pteropus vampyrus	mammals-rodents
A.jamaicensis	XP_036982618.1	Artibeus jamaicensis	mammals-rodents
M.musculus	NP_001369163.1	Mus musculus	mammals-rodents
M.caroli	XP_021008389.1	Mus caroli	mammals-rodents
M.pahari	XP_021044450.1	Mus pahari retinitis	mammals-rodents
R.norvegicus	XP_006257177.2	Rattus norvegicus	mammals-rodents
A.niloticus	XP_034341830.1	Arvicanthis niloticus	mammals-rodents
N.lepida	OBS83715.1	Neotoma lepida	mammals-rodents
T.guttata	XP_030128813.3	Taenopygia guttata	birds
C.kubaryi	XP_041896115.1	Corvus kubaryi	birds
M.alba	XP_038020407.1	Motacilla alba alba	birds
C.ustulatus	XP_032928337.1	Catharus ustulatus	birds
M.ater	XP_036257935.1	Molothrus ater	birds
P.ruficollis	XP_041341292.1	Pyrgilauda ruficollis	birds
N.taczanowskii	XP_041252657.1	Nychostruthus taczanowskii	birds
M.gallopavo	XP_010713307.2	Meleagris gallopavo	birds
A.platyrhynchos	XP_027321017.1	Anas platyrhynchos	birds
A.chrysaetos	XP_040975091.1	Aquila chrysaetos chrysaetos	birds
A.mississippiensis	XP_019341976.1	Alligator mississippiensis	reptiles
M.reevesii	XP_039342994.1	Mauremys reevesii	reptiles
G.evgoodei	XP_030430871.1	Gopherus evgoodei	reptiles
T.carolina	XP_026514975.1	Terrapene carolina triunguis	reptiles
D.coriacea	XP_038273429.1	Dermochelys coriacea	reptiles
C.mydas	XP_037765676	Chelonia mydas	reptiles
T.scripta	XP_034638257.1	Trachemys scripta elegans	reptiles
C.tigris	XP_039181274.1	Crotalus tigris	reptiles
L.agilis	XP_033018352.1	Lacerta agilis	reptiles
R.temporaria	XP_040190364.1	Rana temporaria	amphibians
X.tropicalis	XP_002934955.2	Xenopus tropicalis	amphibians
B.bufo	XP_040261855.1	Bufo bufo	amphibians
R.bivittatum	XP_029463070.1	Rhinatrema bivittatum	amphibians
M.unicolor	XP_030064692.1	Microcaecilia unicolor	amphibians
G.seraphini	XP_033800350.1	Geotrypetes seraphini	amphibians
L.chalumnae	XP_006003825.1	Latimeria chalumnae	fish
D.rerio	NP_001013591	Danio rerio	fish
O.mykiss	XP_036825842.1	Oncorhynchus mykiss	fish
F.heteroclitus	XP_035990871.1	Fundulus heteroclitus	fish
E.cragini	XP_034740219.1	Etheostoma cragini	fish
T.maccoyii	XP_042274143.1	Thunnus maccoyii	fish
B.splendens	XP_029021867.1	Betta splendens	fish
N.whitei	XP_037531000.1	Nematolebias whitei	fish
S.purpuratus	XP792984.3	Strongylocentrotus purpuratus	invertebrates
F.arisanus	JAG83172.1	Fopius arisanus	invertebrates
A.charruanus	KAG5339965.1	Acromyrmex charruanus	invertebrates
P.argentina	KAG5317668.1	Pseudoatta argentina	invertebrates
D.melanogaster	NP_569947	Drosophila melanogaster	invertebrates
M.domestica	XP011296358.1	Musa domestica	invertebrates
C.elegans	NP_498307.1	Cenorabditis elegans	invertebrates

Table S1. Protein sequences used for building the phylogenetic tree of full length ACRC/GCNA orthologs. Accession numbers in NCBI database, species abbreviations and group designations. Related to Main Figure 1A.

Table S2

(A)

Raw data presented in Figure 2A

Expression of acrc in adult zebrafish

**Male**	Set1	Set2	Set3	Set4	Set5	Set6	Mean	SEM
Brain	3.907,6	246,8	3.958,9				2.704,4	1.228,9
Liver	3.907,6	1.366,1	7.671,4				4.315,0	1.831,6
Kidney	1.579,7	272,3	5.502,2				2.451,4	1.571,4
Intestine	5.054,4	1.547,6	4.926,3				3.842,8	1.148,2
Gonads	130.543,7	5.930,1	21.561,8	132.974,2	6.475,2	19.786,6	52.878,6	25.086,8
Eye	1.518,6	44,2	806,5	2.619,1	54,1	907,6	991,7	397,7
Gills	2.892,7	974,5	708,6				1.525,2	688,0
Muscle	483,4	86,0	2.218,8				929,4	654,8

**Female**	Set1	Set2	Set3	Set4	Set5	Set6	Mean	SEM
Brain	1.242,3	841,7	3.341,1				1.808,4	775,0
Liver	1.392,0	3.615,5	2.310,8				2.439,4	645,1
Kidney	4.266,3	21.631,7	9.135,8	9.248,3	23.371,7	14.965,1	13.769,8	3.096,6
Intestine	2.250,9	7.026,6	10.465,8				6.581,1	2.381,9
Gonads	229.534,1	405.826,0	493.211,3				376.190,5	77.546,0
Eye	1.510,3	3.652,4	589,3				1.917,3	907,4
Gills	6.059,4	14.761,7	670,4	13.818,6	7.646,5	692,0	7.274,8	2.499,6
Muscle	1.105,8	3.417,1	506,6				1.676,5	887,3

(B)

Raw data presented in Figure 2B

Expression of sprtn in adult zebrafish

**Male**	Set1	Set2	Set3	Mean	SEM
Brain	11.890,0	688,2	7.668,1	6.748,8	3.266,2
Liver	2.328,7	234,0	2.162,5	1.575,0	672,3
Kidney	2.345,5	281,7	6.779,9	3.135,7	1.917,0
Intestine	753,7	490,9	2.471,1	1.238,5	620,9
Gonads	162.933,2	1.240.998,9	19.168,7	474.366,9	385.556,1
Eye	4.197,2	215,0	664,0	1.692,1	1.259,3
Gills	4.248,8	39.026,5	15.108,9	19.461,4	10.272,6
Muscle	926,7	448,8	3.511,1	1.628,9	951,2

**Female**	Set1	Set2	Set3	Mean	SEM
Brain	5.046,3	16.478,3	8.868,0	10.130,9	3.360,0
Liver	1.842,2	3.128,1	723,3	1.897,8	694,8
Kidney	2.134,7	10.928,0	5.869,3	6.310,7	2.548,0
Intestine	1.477,7	6.322,1	1.061,5	2.953,7	1.688,4
Gonads	58.810,7	54.162,0	87.036,5	66.669,7	10.271,4
Eye	2.241,5	10.597,8	2.068,6	4.969,3	2.814,7
Gills	4.444,1	15.485,2	1.676,1	7.201,8	4.218,1
Muscle	2.460,6	5.781,4	1.338,2	3.193,4	1.333,9

(C)

Raw data presented in Figure 2C

Expression of acrc and sprtn in during zebrafish embryonic development

acrc

Age	Set1	Set2	Set3	Set4	Set5	Mean	SEM
1 hpf	31.923,6	162.029,4				96.976,5	65.052,9
4 hpf	16.026,1	48.758,0	12.103,0	48.900,3	38.244,6	32.806,4	7.916,0
6 hpf	7.375,9	17.188,5				12.282,2	4.906,3
12 hpf	5.098,7	30.288,7				17.693,7	12.595,0
24 hpf	8.475,2	19.062,8	4.756,1	22.953,9	16.655,4	14.380,7	3.377,2
48 hpf	3.616,7	16.196,2	2.138,5	11.368,5	14.553,8	9.574,7	2.851,7
72 hpf	3.733,7	5.973,3				4.853,5	1.119,8

sprtn

Age	Set1	Set2	Set3	Mean	SEM
1 hpf	5.740,2	8.092,9	36.875,0	16.902,7	10.009,0
4 hpf	2.465,9	3.763,7	11.748,4	5.992,7	2.902,0
6 hpf	955,6	4.078,1	4.331,3	3.121,7	1.086,0
12 hpf	1.499,1	2.739,8	6.701,0	3.646,6	1.569,0
24 hpf	981,1	2.001,7	4.496,3	2.493,0	1.044,0
48 hpf	186,0	1.993,5	6.446,6	2.875,4	1.860,0
72 hpf	1.538,8	4.464,2	5.424,9	3.809,3	1.169,0

(D)

Raw data presented in Figure 2D

Expression of *Acrc* in mouse

**Females**				Mean	SEM
Brain	10,1	51,1	31,5	30,9	11,8
Liver	10,8	9,2	1,5	7,2	2,9
Kidney	0,1	0,6	17,3	6,0	5,7
Intestine	0,8			0,8	
Gonads	0,1	0,1		0,1	0,0

**Male**				Mean	SEM
Brain	1,2	23,5	18,0	14,2	6,7
Liver	21,3	0,6	0,2	7,4	7,0
Kidney	1,8	0,3	0,7	0,9	0,4
Intestine	38,6			38,6	
Gonads	4965,0	8128,0	7242,0	6778,3	942,1

Table S2. Raw expression data presented as graphs in Figure 2. MNE (mean normalized expression) values from 3-5 biological replicates, Mean and SEM for **(A)** expression of *acrc* in adult zebrafish male and female tissues; **(B)** expression of *sprtn* in adult zebrafish male and female tissues; **(C)** expression of *acrc* and *sprtn* in zebrafish embryonic development; **(D)** expression of *acrc* in adult mouse male and female tissues.

Table S3

(A)

Genotype	mRNA injected	% Cat. 1	% Cat. 2	% Cat. 3	% Cat. 4	% Cat. 5	mean % alive	SD (% alive)	no. embryos	no. experiments
WT	#	1,0	0,0	0,0	9,8	89,2	99,0	1,4	135	2
acrc $^{rbi5/5}$	#	84,2	15,8	0,0	0,0	0,0	0,0	0,0	54	3
acrc $^{rbi5/+}$	#	56,8	43,2	0,0	0,0	0,0	0,0	0,0	47	2

(B)

Genotype	mRNA injected	#1	#2	# 3	#4	#5	mean % alive	SD (% alive)	no. embryos	no. experiments
WT	#	4,2	0,4	0,3	0,0	95,1	95,4	5,4	372	9
acrc $^{rbi5/5}$	#	89,2	10,6	0,0	0,0	0,3	0,3	0,8	249	9
acrc $^{rbi8/9}$	#	47,4	51,6	0,0	0,0	1,0	1,0	2,1	562	14
acrc $^{rbi5/5}$	+ Acrc-WT	18,4	11,4	26,3	20,3	23,6	70,2	28,0	202	7
acrc $^{rbi8/9}$	+ Acrc-WT	3,2	1,6	5,3	24,2	65,6	95,1	5,4	594	10
acrc $^{rbi5/5}$	+ Acrc-E451A	78,1	21,9	0,0	0,0	0,0	0,0	0,0	63	4
acrc $^{rbi8/9}$	+ Acrc-Sprt	7,4	41,7	33,4	10,9	6,7	50,9	17,6	84	2
acrc $^{rbi5/5}$	+ Acrc-ΔC	18,8	9,6	11,4	31,8	28,5	71,7	30,6	79	2
acrc $^{rbi5/5}$	+ Acrc-ΔSprt	85,5	14,5	0,0	0,0	0,0	0,0	0,0	89	2

(C)

Genotype	mRNA injected	#1	#2	# 3	#4	#5	mean % alive	SD (% alive)	no. embryos	no. experiments
WT	#	3,2	0,2	1,9	3,7	91,1	96,6	5,7	816	16
acrc $^{rbi8/9}$	#	47,4	51,6	0,0	0,0	1,0	1,0	2,1	562	14
acrc $^{rbi8/9}$	+ Acrc-WT	3,2	1,6	5,3	24,2	65,6	95,1	5,4	594	10
acrc $^{rbi8/9}$	+ Acrc-E451A	59,7	40,3	0,0	0,0	0,0	0,0	0,0	206	5
acrc $^{rbi8/9}$	+ Acrc-ΔC	6,4	0,0	10,5	27,6	55,5	93,6	9,9	151	4
acrc $^{rbi8/9}$	+ Acrc-ΔSprt	54,6	45,4	0,0	0,0	0,0	0,0	0,0	143	3
acrc $^{rbi8/9}$	+ DrSprtn	21,7	78,3	0,0	0,0	0,0	0,0	N/A	46	1
acrc $^{rbi8/9}$	+ MmACRC	29,0	71,0	0,0	0,0	0,0	0,0	N/A	31	1
acrc $^{rbi8/9}$	+ HsACRC	49,6	50,4	0,0	0,0	0,0	0,0	0,0	76	2

(D)

Genotype	mRNA injected	#1	#2	# 3	#4	#5	mean % alive	SD (% alive)	no. embryos	no. experiments
WT	#	4,2	0,4	0,3	0,0	95,1	95,4	5,4	372	9
WT	+ Acrc-WT	4,0	0,0	6,8	6,1	83,1	96,0	7,0	63	3
WT	+ Acrc-E451A	5,1	0,0	0,0	0,0	94,9	94,9	7,3	85	2
WT	+ Acrc-ΔC	9,9	0,0	0,0	6,7	83,4	90,1	4,6	68	2
WT	+ Acrc-ΔSprt	4,9	0,0	4,5	0,0	90,6	95,1	1,7	87	2

(E)

Genotype	mRNA injected	#1	#2	# 3	#4	#5	mean % alive	SD (% alive)	no. embryos	no. experiments
WT	#	3,2	0,2	1,9	3,7	91,1	96,6	5,7	816	16
WT	+ Acrc-WT	4,1	0,0	1,9	20,3	73,7	95,9	2,4	289	6
WT	+ Acrc-E451A	9,0	1,5	13,4	20,0	56,0	89,5	7,4	169	3
WT	+ Acrc-ΔC	25,2	0,0	20,3	17,6	36,8	74,8	23,6	78	2
WT	+ Acrc-ΔSprt	12,3	0,9	6,6	30,1	50,1	86,8	8,0	90	2
WT	+ Acrc-Sprt	5,6	2,5	0,0	5,8	86,0	91,9	2,3	118	2
WT	+ HsACRC	5,6	2,9	0,0	0,6	90,9	91,6	8,3	113	2

(F)

Genotype	mRNA injected	#1	#2	# 3	#4	#5	mean % alive	SD (% alive)	no. embryos	no. experiments
WT	#	8,7	1,3	0,0	0,0	90,0	90,0	5,3	119	3
acrc $^{rbi5/5}$	#	76,8	22,6	0,0	0,0	0,6	0,6	1,2	161	4
acrc $^{rbi5/5}$	+ MmACRC	76,4	23,6	0,0	0,0	0,0	0,0	0,0	105	2
acrc $^{rbi5/5}$	+ DrSprtn	74,0	26,0	0,0	0,0	0,0	0,0	0,0	119	3

(G)

Genotype	mRNA injected	#1	#2	# 3	#4	#5	mean % alive	SD (% alive)	no. embryos	no. experiments
WT	#	8,7	1,3	0,0	0,0	90,0	90,0	5,3	119	3
WT	+ MmACRC	9,0	0,0	2,9	40,5	47,6	91,0	7,9	107	2
WT	+ DrSprtn	3,2	0,0	0,9	7,6	88,3	96,8	2,0	78	2

Table S3. Raw data for detailed phenotype analyses at 24 hpf of all rescue experiments in this study. Shown are the mean percentage of each category, the resulting mean percentage of embryos alive (the sum of categories 3, 4 and 5) ± SD, the total number of embryos and number of biological replicates per category. Numbers in green highlight the lack of toxicity (high mean percentage of live embryos) of the rescue constructs when injected into WT; numbers in red highlight the high rescue efficiency (high mean percentage of live embryos) of certain rescue constructs when injected into *acrc* mutants. **(A)** Related to Figure 3G: percentage of survival of $acrc^{rbi5/+}$ heterozygous embryos obtained from crossing homozygous females with WT males, compared to maternal zygotic mutants and WT embryos. **(B)** Related to Figure 4C and Figure S6A and B: rescue experiments with the DrAcrc rescue constructs Acrc-WT, Acrc-E451A, Acrc-SprT, Acrc-ΔC and Acrc-ΔSprT injected in $acrc^{rbi5/rbi5}$, and/or $acrc^{rbi8/rbi9}$ embryos. **(C)** Related to Figures S6B-C: rescue experiments with the following constructs: Acrc-WT, Acrc-E451A, Acrc-ΔC, Acrc-ΔSprT, DrSprtn, MmACRC and HsACRC injected in $acrc^{rbi8/rbi9}$ embryos. **(D)** Related to Figures S8B-C: rescue experiments with the DrAcrc rescue constructs: Acrc-WT, Acrc-E451A, Acrc-ΔC and Acrc-ΔSprT injected in WT embryos, as controls for injections in $acrc^{rbi5/rbi5}$ embryos. **(E)** Related to Figures S8D-E: rescue experiments with the following constructs: Acrc-WT, Acrc-E451A, Acrc-ΔC, Acrc-ΔSprT, Acrc-SprT and HsACRC injected in WT embryos, as controls for injections in $acrc^{rbi8/rbi9}$ embryos. **(F)** Related to Figure 4D and Figure S10A: rescue experiments with the MmACRC and DrSprtn rescue constructs injected in $acrc^{rbi5/rbi5}$ embryos. **(G)** Related to Figures S10B-C: rescue experiments with the MmACRC and DrSprtn rescue constructs injected in WT embryos, as controls for injections in $acrc^{rbi5/rbi5}$ embryos.
